# Brain alarm by self-extracellular nucleic acids: from neuroinflammation to neurodegeneration

**DOI:** 10.1186/s12929-023-00954-y

**Published:** 2023-08-07

**Authors:** Reiner Kunze, Silvia Fischer, Hugo H. Marti, Klaus T. Preissner

**Affiliations:** 1 Institute of Physiology and Pathophysiology, Department of Cardiovascular Physiology, Ruprecht-Karls-University, Im Neuenheimer Feld 326, 69120 Heidelberg, Germany; 2 Department of Biochemistry, Medical School, Justus-Liebig-University, Giessen, Germany; 3 Kerckhoff-Heart-Research-Institute, Department of Cardiology, Medical School, Justus-Liebig-University, Giessen, Germany

**Keywords:** Danger associated molecular patterns, Extracellular ribosomal RNA, Extracellular mitochondrial DNA, Non-coding RNAs, Neutrophil extracellular traps, Pattern recognition receptors, Alzheimer's disease, Parkinson's disease, Multiple sclerosis, Stroke

## Abstract

Neurological disorders such as stroke, multiple sclerosis, as well as the neurodegenerative diseases Parkinson's or Alzheimer's disease are accompanied or even powered by danger associated molecular patterns (DAMPs), defined as endogenous molecules released from stressed or damaged tissue. Besides protein-related DAMPs or “alarmins”, numerous nucleic acid DAMPs exist in body fluids, such as cell-free nuclear and mitochondrial DNA as well as different species of extracellular RNA, collectively termed as self-extracellular nucleic acids (SENAs). Among these, microRNA, long non-coding RNAs, circular RNAs and extracellular ribosomal RNA constitute the majority of RNA-based DAMPs. Upon tissue injury, necrosis or apoptosis, such SENAs are released from neuronal, immune and other cells predominantly in association with extracellular vesicles and may be translocated to target cells where they can induce intracellular regulatory pathways in gene transcription and translation. The majority of SENA-induced signaling reactions in the brain appear to be related to neuroinflammatory processes, often causally associated with the onset or progression of the respective disease. In this review, the impact of the diverse types of SENAs on neuroinflammatory and neurodegenerative diseases will be discussed. Based on the accumulating knowledge in this field, several specific antagonistic approaches are presented that could serve as therapeutic interventions to lower the pathological outcome of the indicated brain disorders.

## Background

Neuroinflammation as a multifactorial process substantially contributes to several neurological diseases such as ischemic stroke, bacterial/viral infections, traumatic brain injury (TBI), and neurodegenerative diseases such as Alzheimer's disease (AD), Parkinson's disease (PD), amyotrophic lateral sclerosis (ALS), or multiple sclerosis (MS) [169, 244, 264, 413]. In the early defense stage, the innate immune system protects against sterile hyperinflammation and microbial infections by the recognition of endogenous alarmins or danger-associated molecular patterns (DAMPs) as well as microbial pathogen-associated molecular patterns (PAMPs), respectively. DAMPs include cytosolic, mitochondrial, or nuclear components such as proteins (e.g. heat shock proteins, histones, high mobility group box protein 1 (HMGB1), cold-inducible RNA-binding protein), carbohydrates (e.g. hyaluronan), nucleic acids (various types of RNA and DNA) and low molecular weight components (e.g. uric acid crystals, ATP, heme). PAMPs include different types of microbial molecules such as bacterial cell wall components as well as viral nucleic acids [26, 114, 276, 482]. These diverse agonists contain specific recognition epitopes that are sensed by pattern recognition receptors (PRRs), which are expressed by a variety of host immune and non-immune cells [26, 132, 482].

A major class of PRRs is defined by cell membrane-expressed or intracellular endosomal Toll-like receptors (TLRs) or cytosolic nucleic acid sensors, the latter being activated predominantly by PAMPs but also by some DAMPs, exhibiting some overlapping specificity and selectivity [132, 199, 262, 459, 482]. Following the binding of a particular agonist at the cell membrane or after endocytosis and degradation in endosomes (particularly relevant for nucleic acid-based PAMPs), the activation of respective PRRs together with the intracellular recruitment of adapter proteins such as myeloid differentiation factor 88 (MyD88) initiate inflammatory immune responses via intracellular signaling pathways. Primarily, these pathways involve the nuclear factor-kappa B (NF-κB) but also other transcription factors that induce the expression and release of cytokines, chemokines and antiviral interferons (IFNs) in immune and non-immune cells [187, 222, 228]. As a consequence, the inflammatory tissue environment is sensitized to help recruiting inflammatory cells (such as neutrophils and monocytes/macrophages) that will remove the pathogens or damaging factors and thereby contribute to tissue repair, resolution of inflammation and cellular homeostasis. Hyperinflammatory conditions may be caused if the defense system remains insufficiently controlled [19, 186].

Numerous self-extracellular nucleic acids (SENAs), including nuclear (nuc) and mitochondrial (mt)DNA, messenger RNA (mRNA), transfer RNA (tRNA), ribosomal RNA (rRNA) and other non-coding RNA species, have been identified as potential DAMPs in a variety of pathophysiological situations [36, 46, 114, 375, 406]. During the previous decade, self-extracellular rRNA (rexRNA), which is predominantly liberated under conditions of tissue damage or cell injury, has been characterized as the primary RNA-type alarmin, but it can also be considered as a damaging factor that contributes to disease progression in ischemic stroke, thrombosis, myocardial infarction, atherosclerosis, rheumatoid arthritis or cancer [37, 110–112, 114, 342]. Besides its multifunctional and disease-promoting potential in sterile inflammatory diseases of several organ systems, the ubiquitous DAMP rexRNA has previously been recognized as a potent adjuvant for PAMPs as well, particularly inducing the activation of TLR2 on macrophages and astrocytes in a synergistic manner [112, 286], thereby serving as relevant sensitizer during microbial infections.

While a variety of non-nucleic acid DAMPs have been recognized and their functional role being partially characterized in the central nervous system (CNS), the contribution of SENAs in neuroinflammatory pathologies remains greatly unexplored, particularly regarding their mechanisms of action. With this review we aim to compile the current knowledge on the involvement of SENAs for the pathophysiology of various neuroinflammatory diseases and their clinical implications. Pertinent open questions in the field such as “Which cell types in the brain do contribute to the release of SENAs with high inflammatory or degenerative potential?” and “Which factors or stress situations can trigger the active or passive release of SENAs in the brain?” will be addressed in the first section with various SENAs and their principal reactions being presented. In the second section we will focus on different receptor types and signaling pathways used by SENAs to transmit their functions. In the third, and major part of this review, we will then collate current scientific knowledge on the role of SENAs in selected neuroinflammatory diseases, namely ischemic stroke, MS, AD, and PD. A further open question: “Which regulatory or antagonistic mechanisms are required or available to dampen or even prevent the pro-inflammatory activation of brain-resident cells and peripheral immune cells?” will be addressed in the perspectives part, where potential therapeutic options to modulate the activity of both adverse and favorable SENAs will be discussed. Finally, in the conclusion section, we put up the question what we can learn from the presented data, and point out some hypotheses and possible directions for future translational research.

## Major types of self-extracellular nucleic acids

Cell-free circulating forms of nucleic acids such as nuclear (nuc)DNA, mitochondrial (mt)DNA, and various species of RNA have been detected in all extracellular fluids, including blood plasma and cerebrospinal fluid (CSF) [272, 373, 383]. Under various pathological conditions such as hypoxia/ischemia, oxidative or metabolic stress, these nucleic acids can leak or are actively released from injured/damaged cells, tumor cells, monocytes/macrophages and other immune cells as well as they are liberated from mast cells during their degranulation reaction into the extracellular environment throughout the body, including the brain [64, 81, 99, 305, 306, 369]. The released exRNA includes several forms of RNA species such as microRNA (miRNA), long non-coding RNA (lncRNA), circular RNA (circRNA), as well as tRNA, rRNA, and mRNA that can be liberated from cells either in free form or in association with proteins, lipids as well as with extracellular vesicles (EVs) [114, 167, 382, 407]. EV-associated exRNAs such as miRNAs are thereby shuttled from one cell type to another to promote the regulation of gene expression at the transcriptional and translational level in the target cell [96, 192, 272]. Yet, a detailed discussion of exRNA-related aspects in this context is beyond the scope of this article, and thus will be only marginally addressed when discussing the role of exRNAs in inflammatory processes during the course of neurological diseases. In the following, the major types of SENAs will be introduced.

### Ribosomal RNA

Circulating rexRNA in body fluids can be liberated in principle from all cell types upon mechanical stress (such as fluid shear stress in blood vessels), hypoxia/ischemia as well as under various cell stimulatory conditions and during tissue injury [58, 111, 203, 324, 407, 477]. RexRNA in isolated form or in complex with proteins or EVs is considered as a common damaging factor in a variety of cardiovascular and non-cardiovascular diseases [305, 306, 436, 446]. Quantitatively, the heterogeneous forms of rexRNA (as ribosomal fragments with a varying degree of ribosomal proteins) are by far the most abundant exRNA species, being detectable in blood plasma, CSF and other body fluids and are considered nucleic acid DAMPs [59, 66, 114].

As to its role as non-typical DAMP, no direct interactions of rexRNA with cell membrane PRRs or other receptors has been recognized so far. Yet, some observations point to the uptake of EV-associated rexRNA fragments that may become recognized by endosomal TLR3 [46], however, unequivocal experimental proofs are missing so far. Nevertheless, rexRNA is able to directly induce the expression and release of several cytokines such as tumor necrosis factor-α (TNF-α) or interleukin-6 (IL-6) in monocytes/macrophages [36, 111], and thereby promotes a robust pro-inflammatory circuit, involving NF-κB-mediated signal transduction. Moreover, rexRNA was found to enhance the activity of pro-inflammatory PAMPs (such as TLR2-ligands) in a synergistic manner both in macrophages and astrocytes [112, 286].

### MicroRNAs

MiRNAs are small ribonucleic acids consisting of 21–25 nucleotides (often folded as hairpins), which primarily act intracellularly as transcriptional and translational regulators by targeting specific mRNAs via base pairing of their untranslated regions to promote e.g. their RNase-dependent degradation. About 70% of the so far identified miRNAs are expressed in the brain. Since various miRNAs are associated with EVs or exosomes that are released from cells upon various stimulations, EV-associated miRNAs can be easily translocated to target cells or tissues, where they are taken up [267]. In the narrower sense, however, miRNAs cannot be considered as an exRNA-DAMP that operates in the extracellular compartment, rather, they facilitate molecular communications between cells. Yet, the functional consequences of miRNA transfer between particular cell types in the CNS for the development of neuroinflammatory or neurodegenerative diseases remains a great challenge for further investigations.

### Circular RNAs

CircRNAs are coding or non-coding RNA molecules, which are characterized by back-splicing and the formation of covalent closed continuous nucleotide loops [265]. These RNAs are defined as single-stranded RNA (ssRNA) formed by head-to-tail splicing of a linear mRNA fragment, which can regulate gene expression at multiple levels as they induce transcription and alternative splicing in the nucleus. Moreover, they serve (together with RNA-binding proteins) as sponges for miRNAs, thereby inhibiting their interactions with the respective mRNA targets in the cytoplasm. Thus, circRNAs are considered as post-transcriptional regulatory elements [313]. In line with these structural properties, including covalently closed loops with neither 5′-3′ polarity nor a poly-adenylated tail, circRNAs are much more stable than linear RNAs and insusceptible to degradation by RNA exonuclease or RNase R [358]. Under pathological conditions, particular circRNAs have been identified in the blood stream as potential biomarkers for certain inflammatory diseases [238].

### Long non-coding RNAs

Another class of ncRNAs entails lncRNAs with a length of more than 200 nucleotides, which are transcribed by RNA polymerase II and processed like protein-coding RNAs [421, 437]. LncRNAs are described to promote apoptosis, angiogenesis, inflammation, or cell death through mechanisms of gene regulation, epigenetically as well as on transcriptional and post-transcriptional levels [28, 94, 131, 322, 437]. As such, lncRNAs are associated with chromatin-modifying enzymes or DNA-binding proteins and thereby mediate activation or silencing of gene transcription [28, 322, 421, 437]. Furthermore, lncRNAs influence nuclear transport mechanisms [20, 468], they modulate gene expression by interfering with the splicing of pre-mRNAs [421], they act upon miRNAs and thereby compete with mRNAs for the binding to their target miRNAs [94, 421, 437], and they influence the assembly of the translation initiation complex [421, 437]. Functional abnormalities in lncRNAs are strongly associated with the development of various inflammatory diseases [105].

### Cell-free extracellular DNA and neutrophil extracellular traps (NETs)

Upon cellular stress, tissue injury or infection, different species of extracellular DNA, particularly cell-free DNA (cfDNA), are detectable in the blood. Under physiological conditions, the level of cfDNA is very low (1–50 ng/ml) due to their degradation, particularly by DNase1 and DNase1-like 3 [7, 403]. Due to mutations in such DNases or due to an impaired apoptotic clearance of cfDNA, autoimmune disorders such as systemic lupus erythematosus (SLE) can develop with the occurrence of anti-DNA antibodies followed by e.g. massive complement activation [349].

Mitochondrial dysfunction leads to the release of mtDAMPs, such as mtDNA, mitochondrial transcription factor A (TFAM), cardiolipin, cytochrome c and other mitochondrial-derived molecules, thereby activating specific inflammatory cascades, collectively referred to as mito-inflammation [293]. Among cfDNAs, mitochondrial exDNA (mexDNA) has been identified as a stable DAMP, being released under conditions of tissue damage and cell death, such as in myocardial infarction, TBI [389] or in response to increased oxidative or metabolic stress [237, 300]. Furthermore, mexDNA is a potent trigger of the innate immunity response due to its bacterial ancestry and the presence of hypo-methylated CpG motifs [300]. Once released into either the cytosol or the extracellular space, mtDNA fragments instigate inflammation via the interaction with PRRs, including TLRs, nucleotide-binding oligomerization domain (NOD)-like receptors (NLRs), or the cyclic GMP/AMP (cGAMP) synthase (cGAS)/stimulator of interferon genes (STING) pathway [123, 300, 462] (see below). In line with the higher resistance of mtDNA towards nuclease-dependent degradation compared with nucDNA, the circulating mexDNA is highly stable and can be detected in body fluids such as plasma and CSF [123].

ExDNA is also a major component of the extracellular decondensed chromatin, designated as NETs, which are released from neutrophils upon activation by various endogenous and exogenous (inflammatory) factors. NETs are generated by NADPH-oxidase-dependent and -independent pathways particularly in neutrophils and mast cells, whereby extrinsic (microbial) as well as intrinsic stimuli (e.g. hydrogen peroxide) or phorbol myristate acetate can induce NETosis [33, 87, 120]. Furthermore, the post-translational modification of histones by the peptidyl-arginine-deiminase-4 (PAD4) is required for loosening the DNA-histone interactions of the chromatin network to promote NETosis [412].

Two major functional areas of NETs have been described:

(a) Upon stimulation of neutrophils, the generated ultra-large scaffold of NETs (composed of the entire decondensed nucDNA, histones, and various antimicrobial proteins and enzymes), serves to catch and kill microbes in the initial immune response [33, 120]. Together with the phagocytic action of macrophages (intracellular killing), the extracellular killing function of NETs thereby serves to protect various organisms from invading pathogens [12, 280].

(b) Activated blood platelets also serve as inducer of NETosis by providing adhesive interactions with neutrophils, culminating in the immediate formation of cellular aggregates from which NETs are released to provoke prothrombotic functions. Besides fibrin, NETs appear to be a major component of the generated venous and arterial thrombi [259, 386]. In fact, in experimental models of thrombosis, the administration of DNase1 significantly prevented or reduced the outcome of thromboembolic diseases [121, 127, 259]. In essence, hardly any inflammatory, cardiovascular or chronic disease is devoid of the generation of NETs, which thereby not only function as a causal disease factor but may also serve as diagnostic or prognostic biomarkers [69, 253].

Meanwhile, not only neutrophils but also mast cells, eosinophils, basophils, macrophages and also microglial cells as the resident immune cell of the CNS have been described to release nucDNA-containing extracellular traps (ETs) in response to various stimuli. Yet, the mechanisms of formation and particular functions of ETs also in an organ-specific context such as the brain are still insufficiently understood and under intensive investigation [335, 416].

## Pattern recognition receptors and self-nucleic acid-mediated inflammatory signal transduction

### Pattern recognition receptors in inflammation

The functional activities of DAMPs and PAMPs in body defense related to the innate immune response are mediated by several receptor types in immune and other cells, designated as PRRs [132, 182, 276, 289, 291, 360]. PRRs were originally described only to recognize specific PAMPs [168], but it is now well accepted that these receptors are also involved in the signal transduction of different DAMPs, including HMGB1, histones, heat shock proteins, as well as SENAs alone or in complex with other components [132, 199, 262, 459, 482]. PRRs such as TLRs are not only expressed by various peripheral immune cells, but also by resident cells of the CNS such as microglia, astrocytes, oligodendrocytes, and neurons, which all participate in the initial immune response against bacterial/viral brain infections and acute CNS injuries (e.g. mechanical traumas, ischemic stroke) [75, 191, 200, 213, 352]. These PRR-expressing cells also play a crucial role in the generation of neuroinflammation in neurodegenerative chronic diseases such as AD, PD, Huntington's disease (HD), ALS, and MS [199, 352] (Table 1).

**Table 1:** Pattern recognition receptors in the CNS and their contribution to neuroinflammatory diseases

Pattern recognition receptors		Cellular sources of expression	Endogenous/artificial RNA/DNA ligands	Associated pathologies
TLRs	TLR2/TLR6 TLR2/TLR1	Astrocytes, microglia, oligodendrocytes, CECs	rexRNA as cofactor	IS: reduced infarct size in TLR2 KO mice [208, 481]; self rexRNA acts as cofactor of TLR2-ligands [112] AD: TLR2 KO in a mouse model of AD aggravates AD pathology (Richard et al. 2008); upregulated TLR2 mRNA in amyloid plaques of patients [117] MS: immune adjuvant properties of TLR2, TLR4, and TLR9 in murine EAE [101] PD: TLR2-mediated signaling in a rat model of PD [279]; impaired TLR2 response in leukocytes of PD patients [63]
	TLR3	Astrocytes, microglia, neurons, oligodendrocytes, CECs	mRNA dsRNA ssRNA poly(I:C), siRNA	IS: inhibition of the TLR3/IRF3-IFNβ-signaling pathway reduced the inflammatory response in a rat model of transient global cerebral ischemia [62]; increased TLR3 and INFβ mRNA expression in PBMCs of stroke patients [78] AD: increased TLR3 mRNA expression in brain tissue of AD patients [388] MS: higher TLR3 expression in immune cells from MS patients [330] PD: poly(I:C) injection in the substantia nigra from rats induced neuroinflammatory processes [76]
	TLR4	Microglia, CECs, neurons	NETs	IS: neuroprotective effects of TLR4 KO in a murine model of transient focal cerebral ischemia [164]; NET formation in the rodent ischemic brain [416] AD: TLR4 dependent upregulation of cytokines in a mouse model of AD [178]; upregulated TLR4 mRNA in amyloid plaques of patients [117] MS: more severe symptoms in TLR4 and TLR9 KO mice subjected to EAE [255]; immune adjuvant properties of TLR2, TLR4, and TLR9 in EAE [101] PD: TLR4-dependent SARS-CoV-2 infection and its possible role in PD pathogenesis [56]
	TLR7/TLR8	Microglia, neurons	miRNA, ssRNA	IS: increased TLR7 and TLR8 expressions are associated with poor outcome and a greater inflammatory response in stroke patients [32] AD: miRNAs can induce TLR7-dependent neurodegeneration [206]; upregulated TLR7 mRNA in amyloid plaques of patients [117] MS: impaired TLR8 expression and signaling in PBMCs from MS patients [179] PD: impaired TLR7/8 response in leukocytes from PD patients [63]; ssRNA-induced TLR7/MyD88-dependent neuronal cell death and neurodegeneration [207]
	TLR9	Microglia, neurons	Oligodeoxynucleotides, unmethylated CpG DNA, DNA	IS: upregulation of TLR9 after cerebral ischemia reperfusion in mice [171] AD: upregulated TLR9 mRNA in amyloid plaques of patients [117]; induction of TLR9 signaling by oligodeoxynucleotides ameliorates AD-related pathology [334] MS: TLR9 and MyD88 are involved in the autoimmune process during EAE [307]; more severe EAE symptoms in TLR4 and TLR9 KO mice [255]; immune adjuvant properties of TLR2, TLR4, and TLR9 in rodent EAE [101] PD: stimulation of the innate immune system by DNA oligodeoxynucleotides [334]
RAGE		Neurons, astrocytes, microglia, vascular cells	DNA, ssRNAs	IS: association of RAGE expression with acute ischemic stroke prognosis [225] AD: targeting of RAGE and TLR4 in experimental models of AD has beneficial effects on the disease progression [294] MS/PD: increased RAGE expressions in brains from MS, AD, and PD patients [353]; RAGE expression, NF-κB-induced cytokine production, and ROS generation are elevated in PD patients [175]
Cytosolic receptors	RIG-1, MDA-5, NLRs, cGAS, inflammasome	Neurons, astrocytes, microglia, vascular cells	dsDNA	IS: inhibition of cGAS ameliorates brain injury after ischemic stroke [215]; NLRP1/3 inflammasome proteins, IL-1β and IL-18 are elevated in brain tissue of mice underwent cerebral ischemia and stroke patients [103] AD: expression of NLRPs, caspases, IL-1β and IL-18 is increased in PBMCs and Aβ plaques of AD patients [384] MS: caspase-1 inhibition prevents inflammasome activation in models of MS [263] PD: higher levels of IL-1β and caspase-1 in serum and brain tissue of PD patients [384]

Aβ: amyloid-β; AD: Alzheimer's disease; CECs: cerebral endothelial cells; cGAS: cyclic GMP/AMP (cGAMP) synthase; EAE: experimental autoimmune encephalomyelitis; IFN: interferon; IS: ischemic stroke; MDA5: melanoma differentiation-associated protein 5; MS: multiple sclerosis; NET: neutrophil extracellular trap; NLR: nucleotide-binding oligomerization domain (NOD)-like receptor; PBMC: peripheral blood mononuclear cell; PD: Parkinson's disease; poly(I:C): polyinosinic:polycytidylic acid; RAGE: receptor for advanced glycation end-products; RIG: retinoic acid-inducible gene; TLR: toll-like receptor

### Toll-like receptors

Within the class of PRRs with a wide variety for recognition of DAMPs and PAMPs, the TLRs exert a key role in both body defense and hyperinflammatory diseases, if left uncontrolled [168, 262, 482]. Cell membrane-expressed TLRs including TLR2, TLR4, TLR5, TLR6, and TLR11 recognize microbial motifs of the pathogen cell wall, whereas TLR3, TLR7, TLR8, TLR9, and TLR13 are expressed on the endosomal compartments and are responsible for the recognition of pathogen-derived exogenous nucleic acids, following their endocytosis and degradation [224, 228, 312]. Since SENAs principally exhibit a much higher degree of post-transcriptional modification than nucleic acid PAMPs, they are largely prevented from recognition by TLRs in order to avoid hyperinflammation and autoimmunity [118].

Double-stranded RNAs (dsRNA) as contained in viruses but also artificial dsRNA such as poly (I:C) as well as fragments of mRNA or exRNA released from necrotic cells were described to activate endosomal TLR3 [42, 185, 241], whereas TLR7/TLR8 preferentially recognize ssRNA [146], and TLR9 shows a preference for recognizing bacterial or viral DNA [273]. The activation of these receptors by self-nucleic acids is very limited, whereas foreign nucleic acids are protected from the ribonucleolytic degradation among others by their capsids unless they are released within the endolysosomal compartment after their cellular uptake via phagocytosis or endocytosis [254].

TLRs belong to the Toll/IL-1 receptor (TIR) family of proteins, and binding of a respective ligand leads to the (hetero-) dimerization of a given TLR, followed by the intracellular recruitment of adaptor proteins. Here, the majority of TLRs use MyD88 as intracellular adaptor protein, which binds to the TIR domain of all TLRs (except endosomal TLR3) to activate downstream signaling pathways, involving IL-1R-associated kinases [146, 360]. The signaling reactions culminate in the phosphorylation of IκB, followed by its ubiquitination and proteasomal degradation, which enables the dissociation of the formerly bound transcription factor NF-κB. NF-κB then translocates into the nucleus and induces the expression/production of inflammatory cytokines such as TNF-α, IL-6, inducible nitric oxide synthase (iNOS), or pro IL-1β [146, 360]. In contrast, TLR3 signals through the TIR domain-containing adaptor inducing IFN-β (TRIF), finally leading to the production of type I IFNs and antiviral immunity-related proteins [360, 424, 425, 441].

### Retinoic acid-inducible gene-1-like receptors

Besides TLRs, cytosolic RNA sensors such as retinoic acid-inducible gene-1 (RIG-1)-like receptors (RLRs), and certain NLRs, or DNA sensors such as absent in melanoma 2 (AIM2) and cGAS contribute to inflammatory responses by various cell types, also in the CNS [191, 323, 408]. The protein family of RLRs include RIG-1, melanoma differentiation-associated protein 5 (MDA5), and laboratory of genetics and physiology 2 (LGP2) [133, 149, 318]. RLRs preferentially recognize dsRNA with different structural features and they are key sensors of virus infections in mediating mainly the transcriptional activation of type I IFNs [318, 323]. All RLRs have a central helicase domain and a so-called carboxy-terminal domain. RIG-1 and MDA5 also contain two amino-terminal caspase activation and recruitment domains (CARD), which interact upon RNA binding, being present in mitochondrial antiviral-signaling protein (MAVS). MAVS serves as the essential adaptor protein initiating the activation of TANK-binding kinase (TBK1) and IκB kinase-ε, which together with NF-κB induce the transcription of genes encoding type I IFNs as well as other immuno-regulatory proteins [133, 149, 318].

### Absent in melanoma 2

AIM2 is another cytosolic sensor that detects double-stranded DNA (dsDNA) of 50–80 bp from foreign species or of self-origin. It triggers the formation of an inflammasome complex resulting in the activation of caspase-1 [158, 159]. The inflammasome is another essential component of the innate immune system mainly involved in NF-κB-dependent expression and production of IL-1β and IL-18. Here, a DAMP- or PAMP-driven initial stimulus provokes a second signal, which is mediated via intracellular receptors such as AIM2-like receptors or NLRs. These events result in the activation of caspase-1, which ultimately drives the assembly of the ultra-large inflammasome protein complex and the maturation and release of IL-1β and IL-18 from their protein precursors [23, 30, 34, 139]. In the CNS, several cells including microglia, neurons, and astrocytes, express components of the inflammasome and may respond to extracellular nucleic acids as well [8]. Moreover, it has been reported that inflammasome proteins in CSF of brain-injured patients could serve as biomarkers of functional outcome, yet, any connection to self-nucleic acids is missing so far [3].

### Cyclic GMP/AMP synthase

Moreover, models of TLR9-independent sterile inflammation indicate the existence of a further cytosolic DNA-sensing pathway [354], which contains cGAS as a cytosolic DNA-sensing PRR [124, 323]. cGAS is a cytoplasmic nucleotidyl-transferase that belongs to the class of template-independent polymerases. Upon dsDNA binding, cGAS catalyzes the conversion of GTP and ATP into 2′3′-cGAMP, followed by the activation of STING, thereby inducing the gene expression of type I IFNs, IFN-stimulated genes, and several other inflammatory mediators, proapoptotic genes and chemokines [74]. cGAS recognizes a broad repertoire of DNA species of both foreign and self-origin (sterile inflammation) [74]. Conclusively, blocking of the cGAS/STING pathway is discussed as a therapeutic regimen in the treatment of several inflammatory diseases [74, 124].

### Receptor for advanced glycation end products

The receptor for advanced glycation end-products (RAGE) belongs to the immunoglobulin superfamily of proteins and was originally described to be recognized and activated by several ligands such as advanced glycated proteins (AGEs), S100 proteins, but also by nucleic acids [27, 163, 344]. In the healthy brain, RAGE is expressed by neurons, astrocytes, microglia, and vascular cells at a low level. However, during various pathological conditions its expression is strongly upregulated in a ligand-dependent manner, and propagates cellular dysfunction in inflammatory diseases [44, 163, 333]. Although AGEs accumulate in the aging brain and could be one of the reasons for age-related diseases like PD, a combined action of DAMPs together with AGEs to provoke engagement/activation of RAGE has not been studied so far.

### Self-nucleic acid-mediated inflammatory signal transduction

Under conditions of cell injury and tissue damage or defects in the intracellular degradation or processing machinery of nucleic acids, substantial amounts of exDNA and exRNA accumulate, resulting in the activation of endosomal TLRs or the aforementioned cytosolic nucleic acid sensors with subsequent activation of the NF-κB signaling pathway [25, 190, 268, 269, 323, 344]. Yet, as mentioned above, several structural features of self-nucleic acids in comparison to nucleic acid-PAMPs prevent the former ones from inducing inflammatory signal transduction reactions. Conversely, due to the insufficient control by extracellular endonucleases or high concentrations of self-nucleic acids that may associate with proteins to induce the production of autoantibodies, autoimmune diseases such as SLE may develop [21, 198, 317]. Accordingly, the RAGE-dependent uptake of complexes containing DNA and the DAMP HMGB1 was demonstrated to stimulate cytokine production in plasmacytoid dendritic cells and B cells [367]. Furthermore, cytosolic RNA- and DNA-sensors might be involved in the recognition of immune responses as well, whereby the stimulation of such nucleic acid receptors are exploited for adjuvant therapies and treatment of non-neurological disorders such as cancer or allergy [273].

Endosomal TLRs also provide major recognition sites for complexes of self-exRNA or self-exDNA fragments with the neutrophil-derived antimicrobial peptide LL37, a C-terminal peptide of human cathelicidin [126, 201]. In fact, in such complexes, LL37 appears to prevent further degradation of exRNA and exDNA. As a consequence, the self-nucleic acid-LL37 complex, but not self-RNA or self-DNA alone, activates TLR7, TLR8, or TLR9, which initiates the autoimmune-inflammatory cascade in e.g. psoriasis, a chronic skin disease [126, 201, 455].

**Fig. 1:**
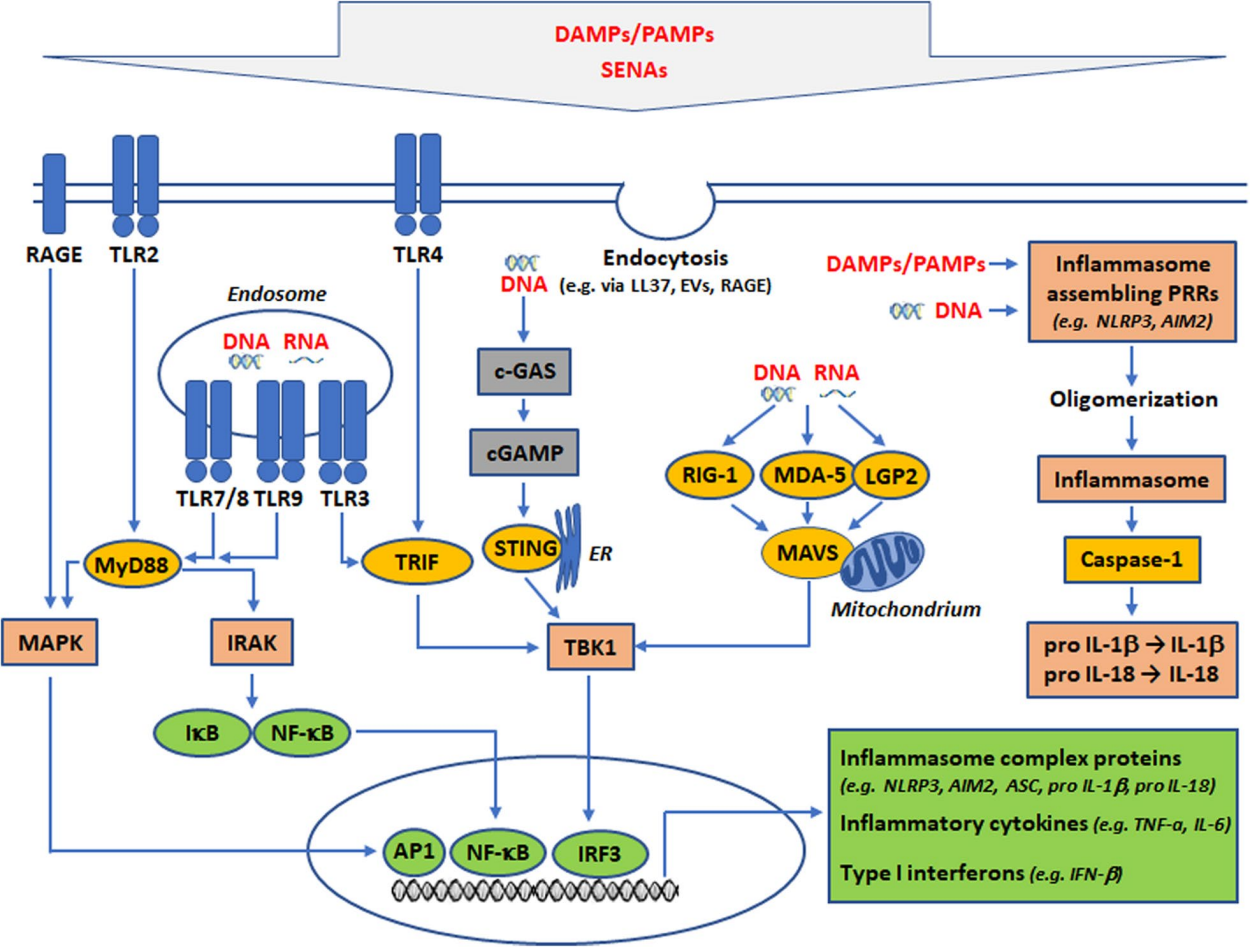
Signaling pathways induced by DAMPs/PAMPs and nucleic acids in the brain. AIM2: Absent in melanoma 2; cGAS: cyclic GMP/AMP (cGAMP) synthase; DAMPs: danger-associated molecular patterns; EV: extracellular vesicle; IRAK: IL-1 receptor-associated kinase; IRF3: interferon regulatory factor 3; LGP2: Laboratory of genetics and physiology 2; MAVS: mitochondrial antiviral-signaling protein; MDA5: melanoma differentiation-associated protein 5; MyD88: myeloid differentiation factor 88; NF-κB: nuclear factor-kappa B; NLR: nucleotide-binding oligomerization domain (NOD)-like receptor; NLRP3: NLR family pyrin domain containing protein 3; PAMPs: pathogen-associated molecular patterns; RAGE: receptor for advanced glycation end-products; RIG-1: retinoic acid-inducible gene-1; SENAs: self-extracellular nucleic acids; STING: stimulator of interferon genes; TLR: Toll-like receptor; TRIF: TIR-domain-containing adaptor-inducing interferon beta; TBK1: TANK-binding kinase

Moreover, SENAs were found to sensitize PRRs for their respective PAMP ligands. As a nucleic acid binding protein, HMGB1 appears to be crucial for the recognition of self-nucleic acids by TLR3, TLR7, and TLR9, whereas no receptor activation occurred in the absence of HMGB1 [427]. Furthermore, a synergistic activation of TLR2 by rexRNA together with TLR2-ligands resulted in the synergistically elevated expression of cytokines [286]. Similarly, complexes of rexRNA with canonical lipopeptide TLR2-ligands but also with HMGB1 caused a pro-inflammatory activation of astrocytes both in vitro and in vivo, indicative for the potential action of self-nucleic acids as sensitizer of brain dysfunction and damage [112]. A graphical illustration of DAMPs, PAMPs, and PRRs and their downstream signaling is presented in Fig. 1.

## Self-extracellular nucleic acids in neuroinflammatory diseases

### Ischemic stroke

#### Etiology and pathogenesis of ischemic stroke

Stroke is the major cause of adult physical disability and one of the leading causes of mortality worldwide, accounting for 7.08 million deaths in 2020. Ischemic stroke is responsible for approximately 87% of all strokes, while hemorrhagic stroke accounts for 13% [374]. Ischemic stroke occurs as a result of interruption of blood flow to the brain due to thrombotic and embolic events. Despite the huge global burden of ischemic stroke, intravenous thrombolysis with recombinant tissue plasminogen activator (rt-PA) and mechanical thrombectomy are the only evidence-based treatment options approved for acute ischemic stroke [321]. However, the uptake of intravenously administered rt-PA is limited by a clinically challenging diagnosis, short therapeutic time window and numerous contra-indications. Similarly, mechanical thrombectomy is only indicated for patients with acute ischemic stroke due to a large cerebral artery occlusion in the anterior circulation, and needs to be achieved within 6 to 24 h (for certain patients) after stroke onset [362]. Therefore, there is an urgent need to better understand pathological cellular and molecular mechanisms in ischemic stroke to develop novel therapeutic perspectives that can protect and recover salvageable brain tissue.

The onset of cerebral ischemia initiates a complex cascade of several interrelated and overlapping pathological mechanisms. The first event of the ischemic cascade is the reduction of oxygen and glucose, which leads to a failure to produce high-energy metabolites to maintain the cellular homeostasis. The involved processes include ionic imbalance, excitotoxicity, calcium overload, cytotoxic and vasogenic edema, peri-infarct depolarization, oxidative and nitrosative stress, cell death, BBB disruption, and inflammation [256]. Immediately after onset of cerebral ischemia, injured and dying neurons release DAMPs to be involved in the activation of brain-resident cells, including microglia, astrocytes, and endothelial cells. Upon M1-like polarization of microglia, reactive astrocytes and activated endothelial cells release pro-inflammatory cytokines, matrix metalloproteinases and reactive oxygen species (ROS), which cause the loss of the BBB integrity [169, 181]. Moreover, pericytes and astrocytic endfeets are lifted from the basement membrane, which further weakens the BBB allowing circulating leukocytes to infiltrate the cerebral parenchyma, where they produce pro-inflammatory factors and exacerbate tissue injury. In the delayed subacute phase, microglia/macrophage switch to an M2-like (anti-inflammatory) phenotype results in the clearance of cellular debris, and, by expressing anti-inflammatory mediators and neurotrophic factors, promotes glial scar formation as well as BBB repair, neurogenesis, oligodendrogenesis, and angiogenesis [169, 181].

#### The role of extracellular RNAs in the neuroinflammatory cascade after ischemic stroke

Various species of exRNA are expressed and become released from brain microvascular endothelial cells, thereby participating in the regulation of the blood–brain barrier (BBB) permeability [109, 431]. We have previously demonstrated in a rat stroke model that self-rexRNA did aggravate ischemic injury by inducing vascular permeability via VEGF [110, 113], and that pretreatment of animals with RNase1 resulted in vessel protection accompanied by reduced edema formation as well as a smaller infarct volume [110, 387] (Table 2). We also reported that neurons respond to hypoxia/ischemia or glutamate excitotoxicity with the release of rexRNA. Although most rexRNA is probably liberated into the extracellular space in a passive manner by necrotic cell death, an active calcium-dependent release of rexRNA by structurally intact neurons was observed as well [112]. While low-dose rexRNA alone had no pro-inflammatory activity on astrocytes, a prominent TLR2/NF-κB-dependent signaling mechanism was achieved in the presence of either Pam2CSK4 (a synthetic PAMP molecule that mimics bacterial infection) or HMGB1 (the most abundant DAMP, liberated in ischemic brain tissue) [112]. Conclusively, self-exRNA may act as an essential sensitizer or adjuvant to engage the binding of endogenous DAMPs to their cognate receptors to trigger sterile inflammation during the course of ischemic stroke.

**Table 2:** Therapeutic potential of DNase/RNase treatment in preclinical animal models of neurological diseases

Disease	Experimental model	Drugs	Time of delivery	Outcomes	Potential mechanisms	References
IS	C57BL/6 mice Permanent MCAO/24 h survival	rhDNase1 50 µg/animal ip and 10 µg/animal iv	10 min after MCAO	Infarct volume reduced Functional outcome improved	Degradation of NETs in brain parenchyma	[296]
	C57BL/6 mice 1 h MCAO/24 h RP	rhDNase1 2.5 mg/kg	Immediately before MCAO	Plasma NET levels reduced Brain infarct size decreased Neurological and motor function improved	Disruption of NETs	[80]
	C57BL/6 mice 2 h MCAO/24 h RP	rhDNase1 50 µg/animal ip 10 µg/animal iv 50 µg/animal ip	15 min before MCAO 5 min before RP 10 h after RP	Infarct volume reduced	Prevention of NETosis probably through regulating vWF and PAI-1	[460]
	C57BL/6 mice Photothrombotic MCAO/24 h survival	rhtPA 10 mg/kg iv rhDNase1 50 µg/animal ip and 10 µg/animal iv 50 µg/animal ip	2 h after MCAO 15 min before tPA 13 h after MCAO	Reduced BBB breakdown, cerebral hemorrhage neurological deficits in tPA/DNase-treated mice compared to tPA-treated mice	Suppression of tPA-induced upregulation of cGAS-STING and type 1 IFN signaling by clearance of NETs	[397]
	C57BL/6 mice Permanent MCAO/3 or 14 days survival	rhDNase1 50 µg/animal ip and 10 µg/animal iv 50 µg/animal ip	24 h or 7 d after MCAO every 12 h until day 3 or day 14	BBB breakdown reduced Neovascularization and vascular remodeling increased	Prevention of stroke-induced STING-mediated production of IFN-β by disruption of NETs	[184]
	C57BL/6 mice Photothrombotic MCAO/24 h survival	rhDNase1 50 µg/animal ip and 10 µg/animal iv	3 h after MCAO	Vessel recanalization improved Infarct volume reduced Functional outcome improved	Disruption of NETs	[297]
	C57BL/6 mice 1 h MCAO/24 h RP	rhDNase1 50 µg/animal ip 10 µg/animal iv 50 µg/animal ip rhDNase1 50 µg/animal ip and 10 µg/animal iv 50 µg/animal ip	15 min before MCAO 5 min before RP 11 h after RP 1 h after RP 13 h after RP	Infarct volume reduced Functional outcome improved Infarct volume reduced Functional outcome not altered	Degradation of extracellular chromatin	[70]
	Wistar rats 90 min MCAO/24 h RP	RNase1 (bacterial) 13–3375 µg/kg iv	10 min before MCAO	Infarct volume reduced Vasogenic edema decreased Motor impairment improved	n.d	[387]
	Wistar rats 90 min MCAO/24 h RP	RNase1 (bacterial) 42 µg/kg iv	Immediately before MCAO	Infarct volume reduced BBB hyperpermeability and vasogenic edema decreased	Blockage of VEGF-mediated disintegration of interendothelial tight junctions	[110]
ICH	Sprague–Dawley rats 200 µl autologous arterial blood at 20 µl/min icv 7 days survival	rhtPA 20 µg/animal icv rhDNase1 2000 IU/animal icv	1 h after hematoma placement	Reduced ventricular dilation, neurological impairment astrogliosis in tPA/DNase-treated mice compared to tPA-treated mice	Potentiation of tPA-induced fibrinolysis by degradation of clot-associated cell-free DNA	[419]
	Sprague–Dawley rats 100 µl autologous arterial blood at 10 µl/min icv 3 days survival	rhtPA 20 µg/animal icv rhDNase1 2000 IU/animal icv	1 h after hematoma placement	Improved hematoma resolution, brain swelling and neurological deficits in tPA/DNase-treated mice compared to tPA-treated mice	Potentiation of tPA-induced fibrinolysis by disintegration of NETs	[361]
SAH	C57BL/6 mice Endovascular filament perforation model 1 day survival	rhDNase1 50 µg/animal ip and 10 µg/animal iv	3 h after SAH	NETs in brain parenchyma decreased Brain swelling reduced Neurological dysfunction improved Neuroinflammatory response alleviated	Disruption of NETs	[444]
	C57BL/6 mice Endovascular filament perforation model 1, 7 or 14 days survival	RNase-A (bovine) 42 µg/kg iv 42 µg/kg iv	Perioperative every 3 days until day 7 or day 14	Accumulation of NETs reduced	n.d	[119]
TBI	C57BL/6 mice Controlled cortical impact model 24 h survival	RNase-A (bovine) 20–180 µg/kg ip	0.5 and 12 h after TBI	Lesion volume reduced BBB breakdown and vasogenic edema decreased Functional outcome not altered	n.d	[196]
	CD-1 mice Controlled cortical impact model 24 h or 2 months survival	rhDNase1 5 mg/kg iv	1 h after TBI	Vasogenic edema reduced Cerebral perfusion improved Acute and chronic neurobehavioral outcomes improved	Degradation of circulating and CNS-infiltrated NETs	[376]
POCD	C57BL/6 mice Unilateral nephrectomy 1, 3 or 7 days survival	RNase-A (bovine) 500 µg/animal sc 200 µg/animal ip 500 µg/animal sc	0.5 h prior to right before 1 h after nephrectomy	Cognitive impairment attenuated Inflammatory cytokine expression in serum and hippocampus reduced Apoptosis in hippocampus decreased	n.d	[45]
ALS	SOD1^G93A^ C57B6.Cg-Tg mice	rhANG (RNase5) 1 µg/animal ip	3×/week from PND 90 until PND 115	Reduced spinal cord motoneuron loss and vascular network regression, delayed motor dysfunction and improved survival of SOD1^G93A^ mice	n.d	[60]
	SOD1^G93A^ C57B6.Cg-Tg mice	rhRNase4 10 µg/animal ip	1×/week from 11 weeks of age until death	Slowed weight loss and enhanced neuromuscular function of SOD1^G93A^ mice	n.d	[216]

ANG: angiogenin; ALS: amyotrophic lateral sclerosis; BBB: blood–brain barrier; cGAS: cyclic GMP-AMP synthase; CNS: central nervous system; ICH: intracerebral hemorrhage; icv: intracerebroventricular; IFN: interferon; ip: intraperitoneal; IS: ischemic stroke; iv: intravenous; MCAO: middle cerebral artery occlusion; n.d.: not determined; NET: neutrophil extracellular trap; PAI-1: plasminogen activator inhibitor 1; PND: postnatal day; POCD: postoperative cognitive dysfunction; RP: reperfusion; SAH: subarachnoid haemorrhage; sc: subcutaneous; STING: stimulator of interferon genes; tPA: tissue plasminogen activator; TBI: traumatic brain injury; VEGF: vascular endothelial growth factor; vWF: von Willebrand factor

Further experimental evidence has demonstrated that regulatory ncRNAs are involved in many aspects of the pathogenic mechanisms that underlie the tissue damage following stroke, including excitotoxicity, oxidative stress, neuroinflammation, BBB damage and apoptosis as well as aspects of post-stroke recovery including neurogenesis and angiogenesis. Apart from intracellular, functionally active ncRNAs, including housekeeping RNAs (rRNA, tRNA, small nuclear and nucleolar RNAs) as well as miRNAs, lncRNAs and circRNAs, circulating extracellular ncRNAs have been proposed as potential clinical stroke biomarkers with regard to diagnosis, prognosis or disease severity (ncRNAs with proven prognostic value in human stroke patients are summarized in Table 3). Several selected examples of regulatory ncRNAs that either augment or mitigate the neuroinflammatory response to ischemic stroke are discussed below. For a more comprehensive overview the reader is referred to recent review articles in the field [183, 211, 231, 381, 411].

**Table 3:** Summary of SENAs associated with disease severity and prognosis of ischemic stroke in human patients

SENA	Study population	Sample type	RNA/DNA name	Findings	References
miRNA	60 HC 112 IS	Peripheral blood leukocytes	miR-210	Downregulated in IS patients Expression inversely correlated with disease severity	[445]
	27 IS	Plasma	miR-941, miR-449b, miR-581	Expression positively correlates with motor function recovery	[95]
			miR371-3p, miR-524, miR-520 g, miR-1255A, miR-453, miR-583	Expression negatively correlates with motor function recovery	
	329 IS	Plasma	miR-150-5p	Expression inversely correlates with mortality within 3 months after stroke	[332]
	59 HC 58 IS	Peripheral blood leukocytes	miR-29b	Downregulated in IS patients Expression is inversely associated with disability level and brain infarct volume	[399]
	23 HC 33 IS	Plasma	miR-16	Upregulated in IS patients Expression positively correlates with disease severity	[366]
	102 HC 128 IS	Serum	miR-146b	Upregulated in IS patients Expression positively correlates with disability level and infarct volume	[53]
	84 IS	Plasma	miR-124-3p, miR-125b-5p, miR-192-5p	Expression positively correlates with unfavorable outcome at 3 months after stroke	[154]
	94 IS	Plasma	miR-125b-5p, miR-206	Expression positively correlates with disability level and infarct volume	[155]
	58 IS	Plasma	miR-21-5p, miR-206, miR-3123	Expression correlates with the risk of hemorrhagic transformation	[471]
	84 IS	Plasma	miR-124-3p	Expression positively correlates with disease severity and mortality within 3 months after stroke	[315]
			miR-16	Expression negatively correlates with disease severity and mortality	
	38 HC 76 IS	Serum	miR-132	Upregulated in IS patients Expression positively correlates with post-stroke cognitive impairment	[162]
	21 HC 21 IS	CSF	miR-9-5p, miR-9-3p, miR-124-3p, miR-128-3p	Expression positively correlates with infarct volume	[350]
	110 HC 106 IS	Plasma	miR-126, miR-378	Declined in IS patients Expression inversely correlates with disease severity	[177]
			miR-222	Elevated in IS patients Expression positively correlates with disease severity	
	33 HC 50 IS	Serum-derived exosome	miR-223	Upregulated in IS patients Expression positively correlates with disease severity and poor outcome at 3 months after stroke	[51]
	66 HC 65 IS	Serum-derived exosome	miR-9, miR-124	Increased in IS patients Expression positively correlates with disease severity, infarct volume and serum level of IL-6	[170]
	42 HC 177 IS	Serum	miR-23b-3p, miR-29b-3p	Increased in IS patients Expression positively correlates with disease severity	[414]
lncRNA	189 HC 189 IS	Peripheral blood leukocytes	MIAT	Upregulated in IS patients Expression positively correlates with disease severity, infarct volume as well as unfavorable functional outcome at 3 months after stroke and 3-month mortality	[478]
	210 HC 210 IS	Plasma	NEAT1	Elevated in IS patients Expression positively correlates with disease severity and pro-inflammatory factor levels Expression is inversely associated with 36-month recurrence‐free survival	[214]
	60 HC 120 IS	Plasma	GAS5	Upregulated in IS patients Expression positively correlates with disease severity and pro-inflammatory factor levels Expression is inversely associated with 36-month recurrence‐free survival	[102]
	320 HC 320 IS	Plasma	lnc-ITSN1-2	Increased in IS patients Expression is positively associated with disease severity and levels of pro-inflammatory cytokines Expression is negatively associated with 36-month recurrence‐free survival	[465]
	120 HC 241 IS	PBMC	lnc-ZFAS1	Downregulated in IS patients Expression is inversely associated with disease severity and levels of pro-inflammatory cytokines Expression positively correlates with 36-month recurrence‐free survival	[390]
	120 HC 120 IS	Plasma	MALAT1	Reduced in IS patients Expression is inversely associated with disease severity and levels of pro-inflammatory cytokines Expression positively correlates with 42-month recurrence‐free survival	[320]
	153 HC 215 IS	Peripheral blood leukocytes	MEG3	Upregulated in IS patients Expression positively correlates with disease severity, infarct volume as well as unfavorable functional outcome after 6 months and 6-month mortality	[394]
	160 HC 160 IS	Blood CD4+ T cells	UCA1	Elevated in IS patients Expression positively correlates with disease severity, pro-inflammatory factor levels and Th17 cell proportion Expression is inversely associated with 36-month recurrence‐free survival	[319]
	25 HC 36 IS	Plasma	H19	Increased in IS patients Expression positively correlates with disease severity and TNF-α levels	[393]
	95 HC 103 IS	Plasma	NORAD	Upregulated in IS patients Expression positively correlates with disease severity, MMP9 levels and mortality within 3 months after stroke	[226]
	125 HC 126 IS	Plasma	ANRIL	Decreased in IS patients Expression is inversely associated with disease severity and pro-inflammatory factor levels	[106]
	51 HC 181 IS	Serum	NEAT1	Increased in IS patients Expression is inversely associated with disease severity	[9]
			GAS5	Reduced in IS patients Expression negatively correlates with disease severity	
			HOTAIR	Declined in IS patients Expression positively correlates with disease severity	
	60 HC 120 IS	PBMC	SNHG16	Downregulated in IS patients Expression is inversely associated with disease severity and levels of pro-inflammatory factors	[418]
	215 HC 215 IS	Plasma	HULC	Upregulated in IS patients Expression is positively associated with disease severity and levels of pro-inflammatory cytokines Expression negatively correlates with 36-month recurrence‐free survival	[50]
circRNA	100 HC 200 IS	Plasma	circFUNDC1, circPDS5B, circCDC14A	Elevated in IS patients Expression positively correlates with unfavorable functional outcome at 3 months after stroke	[486]
	160 HC 160 IS	PBMC	circHECTD1	Increased in IS patients Expression is positively associated with disease severity and levels of pro-inflammatory cytokines, but inversely correlates with 46-month recurrence‐free survival	[299]
cfDNA	91 IS	Plasma	cfDNA	Concentration positively correlates with unfavorable functional outcome at 3 months and 3-months mortality	[135]
	26 IS	Plasma	cfDNA	Concentration is positively associated with disease severity and poor outcome at 3 months	[378]
	54 IS	Plasma	cfDNA	Concentration positively correlates with disease severity and unfavorable outcome at 3 months	[379]
	50 HC 50 IS	Plasma	nucDNA	Concentration positively correlates with disease severity	[372]

cfDNA: cell-free DNA; circRNA: circular RNA; CSF: cerebrospinal fluid; GAS5: growth arrest-specific 5; HC: healthy control; HECTD1: HECT domain E3 ubiquitin protein ligase 1; HOTAIR: HOX transcript antisense intergenic RNA; IS: ischemic stroke; lnc-ITSN1-2: long non‐coding RNA intersectin 1‐2; lncRNA: long non-coding RNA; MALAT1: metastasis-associated lung adenocarcinoma transcript 1; MEG3: maternally expressed gene 3; MIAT: myocardial infarction-associated transcript; miRNA: microRNA; MMP9: matrix metalloproteinase-9; nucDNA: nuclear DNA; NEAT1: nuclear enriched abundant transcript 1; PBMC: peripheral blood mononuclear cells; SENA: self-extracellular nucleic acid; SNHG16: small nucleolar RNA host gene 16; TNF-α: tumor necrosis factor-alpha; UCA1: urothelial carcinoma-associated 1; ZFAS1: zinc finger antisense 1

#### MicroRNAs and stroke-associated neuroinflammation

Accumulating evidence indicates that particular miRNAs play an important role in post-ischemic inflammatory responses (for an overview see also [218]). Upon cerebral ischemia/reperfusion (I/R) injury in mice, the expression of miR-455-5p in the brain parenchyma and respective levels in peripheral blood are decreased [453]. Intracerebral pretreatment with agomir-455-5p, a miR-455-4p mimic, decreased the infarct volume, enhanced BBB integrity, and improved the neurological function, whereas administration of the miR-455-5p antagonist antagomiR-455-5p amplified these pathogenic processes [453]. Moreover, miR-455-5p agonism alleviated stroke-induced microglia activation and release of inflammatory factors at least partly by downregulation of C-C chemokine receptor type 5 [453]. Similarly, in a murine model of ischemic stroke, intracerebral application of miR-671-5p agomir alleviated tissue injury, functional deficits and neuroinflammatory processes by directly targeting the NF-κB mRNA expression [77].

Furthermore, let-7c-5p levels were demonstrated to be decreased in patients with acute stroke but also in mice that underwent I/R injury. Intracerebral let-7c-5p overexpression reduced neuroinflammation, infarct volume and functional deficits after ischemic stroke in mice as well [282]. Accordingly, overexpression in vitro of let-7c-5p suppressed the expression of pro-inflammatory mediators in microglia activated by either lipopolysaccharide (LPS), by oxygen–glucose deprivation/reoxygenation (OGD/R) or the exposure to conditioned medium obtained from OGD-treated neurons. Let-7c-5p inhibited the pro-inflammatory activation of microglia via the direct targeting of caspase-3 [282].

As another example of anti-inflammatory miRNAs in the context of ischemic stroke, the expression of miR-210 is substantially upregulated in astrocytes of human brain tissue from white matter stroke patients as well as in primary human fetal astrocytes, exposed to a combination of hypoxic and inflammatory stress in vitro [189]. The transfection with miR-210-mimics increased glycolysis, enhanced lactate export, and promoted an anti-inflammatory transcriptional and translational signature in human astrocytes [189]. In contrast, the pre- and post-stroke treatment with a miR-210 inhibitor in mice significantly decreased cerebral infarction, behavioral deficits, expression of pro-inflammatory cytokines, microglial activation, and macrophage infiltration [160].

Furthermore, the expression of pro-inflammatory miR-3473b is upregulated in the cortex and striatum of mice following experimental stroke. An intracerebroventricular injection of the miR-3473b antagomir prior to stroke remarkably attenuated the ischemia-induced expression of miR-3473b and pro-inflammatory factors, decreased infarct volume and sensorimotor impairment [398]. Complementary in vitro experiments revealed that miR-3473b triggers the pro-inflammatory activation of microglia via inhibition of suppressor of cytokine signaling 3 (SOCS3), an intracellular, cytokine-inducible protein that inhibits cytokine signaling in numerous cell types [398].

Finally, in a mouse model of ischemic stroke, global genetic ablation of pro-inflammatory miR-155 reduced the extent of brain tissue damage and improved neurobehavioral impairments [405]. Intracerebral overexpression of miR-155 further enhanced the expression of pro-inflammatory cytokines in the ischemic brain by upregulating TLR4 and NF-κB expression as well as downregulating SOCS1 and MyD88, whereas miR-155 knockout abrogated the effects of cerebral ischemia on the TLR4/NF-κB/MyD88/SOCS1 axis [405].

Conclusively, inducing anti-inflammatory miRNAs or suppressing pro-inflammatory miRNAs could be a therapeutic strategy to ameliorate brain tissue damage following ischemic stroke. However, much work remains to be done in deciphering disease-specific miRNA-mRNA interactions, developing efficient systems for the targeted delivery of miRNA-based therapeutics across the BBB and in determining therapeutic windows and modes of treatment.

#### Long non-coding RNAs and stroke-associated neuroinflammation

A large number of studies have illustrated that various lncRNAs are closely associated with the regulation of inflammation and microglial activation in cerebral ischemia (summarized in [292]). For example, the pro-inflammatory lncRNAs nuclear paraspeckle assembly transcript 1 (NEAT1), functional intergenic RNA repeat element (FIRRE), Gm4419, and small nucleolar RNA host gene 14 (SNHG14) are upregulated in microglia exposed to OGD/R, and promote microglial activation via different mechanisms [48]. NEAT1 promotes microglial activation via the Wnt/β-catenin signaling pathway [143], whereas the FIRRE and NF-κB pathway forms a positive feedback loop promoting activation of the NLR family pyrin domain containing protein 3 (NLRP3) inflammasome [442]. Similarly, Gm4419 facilitates microglial activation upon ischemic stress via activation of the NF-κB pathway [404]. SNHG14 increases the expression of cytosolic phospholipase A2 via competitively interacting with miR-145-5p, which contributes to activation of microglial cells in ischemic stroke [311].

In contrast, anti-inflammatory lncRNA SNHG8 is downregulated in brain tissue of mice that underwent experimental stroke as well as microglia exposed to OGD/R. SNHG8 overexpression attenuated the microglial inflammatory response by regulating the miR-425-5p/sirtuin 1 (Sirt1)/NF-κB axis [368].

The anti-inflammatory lncRNA metastasis-associated lung adenocarcinoma transcript 1 (MALAT1) has been shown to be upregulated in microglia and neurons during ischemic stroke, resulting in enhanced sponging of the targeted miRNAs [40, 449]. For example, the MALAT-mediated decrease of miR-375 and miR-181c-5p causes enhanced expression of phosphodiesterase 4D and HMGB1, respectively, aggravating the extent of neuroinflammation during acute stroke [40, 449].

Compared with healthy controls, the level of lncRNA SNHG4 in CSF samples of patients with acute ischemic stroke as well as in microglia of mice subjected to I/R injury was remarkably downregulated, whereas the expression of miR-449c-5p went strongly up [461]. Both, overexpression of SNHG4 and knockdown of miR-449c-5p inhibited the expression of pro-inflammatory cytokines in microglia and promoted the expression of anti-inflammatory factors in microglia at least partly through activation of signal transducer and activator of transcription 6 (STAT6) [461].

Furthermore, lncRNAs also influence the polarization of microglia following ischemic stroke. Gain- and loss-of-function experiments provided convincing evidence that pro-inflammatory lncRNA taurine upregulated 1 (TUG1) and rhabdomyosarcoma 2-associated transcript (RMST) trigger microglial polarization towards a pro-inflammatory (M1-like) phenotype by activation of the NF-κB pathway via competitive interaction with miR-145a-5p and heterogeneous nuclear ribonucleoprotein K (hnRNP K), respectively [357, 391].

Similarly, lncRNA H19 is significantly increased in microglia exposed to I/R in vitro and in vivo and promotes neuroinflammation by driving histone deacetylase 1 (HDAC1)-dependent M1 microglial polarization [393].

In contrast, anti-inflammatory lncRNA Nesp-antisense (Nespas) reduces the polarization of microglia toward pro-inflammatory phenotype through direct interaction with transforming growth factor-β-activated kinase 1 (TAK1), which suppresses the TAK1-mediated activation of the NF-κB pathway [79].

A previous study demonstrated that lncRNA 1810034E14Rik is downregulated in OGD-exposed microglia. The overexpression of 1810034E14Rik decreased the infarct volume and production of pro-inflammatory factors in mice subjected to ischemic stroke in vivo, and promoted polarization of OGD-exposed microglia toward anti-inflammatory M2 phenotype in vitro via inhibiting the NF-κB pathway [463].

Upon cerebral ischemia rapid activation of brain-resident microglia, predominantly by DAMPs released from injured and dying cells, leads to a massive liberation of pro-inflammatory cytokines and chemokines, which substantially contribute to the recruitment and infiltration of circulating immune cells into the ischemic area, exhibiting both detrimental and beneficial effects on the outcome of stroke [169]. In this regard, macrophage contained LCP1 related pro-inflammatory lncRNA (Maclpil) was demonstrated to be highly expressed in pro-inflammatory monocyte-derived macrophages but not in microglia-derived macrophages purified from ischemic mouse brain three days after stroke [401]. Exposure of bone marrow-derived macrophages in vitro to either pro-inflammatory stimuli or OGD revealed that Maclpil triggers cell polarization towards a pro-inflammatory phenotype through lymphocyte cytosolic protein 1 (LCP1) [400, 401]. Also, adoptive transfer of Maclpil silenced macrophages or systemic silencing of Maclpil reduced ischemic brain infarction, improved functional deficits and attenuated the accumulation of monocyte-derived macrophages, CD4+ T cells, and CD8+ T cells in the ischemic hemisphere without affecting microglia cellularity [400, 401].

Despite the emerging importance of lncRNAs in ischemic stroke, being unraveled in a growing number of preclinical studies, further investigations are needed to elucidate lncRNA biological functions to accelerate the progress of lncRNA-based therapeutics against stroke.

#### Circular RNAs and stroke-associated neuroinflammation

Recent studies have proposed that circRNAs exert a central effect in neuroinflammation caused by acute cerebral ischemia. In blood samples from patients with acute stroke as well as in brain tissue from mice subjected to ischemic stroke, circ_0000831 levels were strongly decreased as compared to healthy controls [161]. Intracerebral overexpression of circ_0000831 in mice substantially ameliorated infarct volume, cell apoptosis, BBB dysfunction, vasogenic edema formation, oxidative stress and neuroinflammation [161]. Mechanistically, circ_0000831 overexpression repressed apoptosis and the release of pro-inflammatory factors induced by OGD in microglia via activation of the adiponectin receptor 2/peroxisome proliferator-activated receptor-γ (PPARγ) axis by downregulating miR-16-5p [161]. Consistently, the beneficial effects on the outcome of murine ischemic stroke evoked through circ_0000831 overexpression were almost completely prevented by the intracerebral knockdown of PPARγ [161].

In mice subjected to ischemic stroke, the expression of circ_CDC14A increased in circulating neutrophils within hours. Two to three days upon stroke, circ_CDC14A levels also increased in astrocytes colocalized with neutrophils that infiltrated into the peri-infarct cortex, indicating an intercellular transfer of circ_CDC14A from infiltrating neutrophils to resident astrocytes [485]. A selective knockdown of circ_CDC14A expression in peripheral blood cells, but not in brain tissue, evoked anti-inflammatory effects as it inhibited the activation of astrocytes in the peri-infarct cortex, increased the N2/N1 ratio of neutrophil populations in the ischemic brain, reduced the infarct size, and improved functional impairment und post-stroke survival [485].

Furthermore, in microglial cells exposed to pro-inflammatory stimuli, the overexpression of circ_Dlgap4 promoted the decay of pro-inflammatory cytokine mRNAs by interacting with AU-rich element/poly(U)-binding/degradation factor 1 (AUF1) [229]. Accordingly, the intracerebral overexpression of anti-inflammatory circ_Dlgap4 in a mouse model of ischemic stroke reduced neuroinflammation, brain tissue damage and neurobehavioral deficits, all of which were reversed by the intracerebral knockdown of AUF1 [229].

Taken together, although recent studies in animals provided the first evidence that certain circRNAs play a pivotal role in neuroinflammation and other pathological processes in the course of ischemic stroke, the clinical evaluation of circRNAs as potential diagnostic biomarker and therapeutic target for stroke is still in its early stage.

#### The importance of extracellular DNA for neuroinflammatory processes in ischemic stroke

Microglia, as the major resident immune cell in the CNS, has emerged as a key mediator of neuroinflammation in the course of ischemic stroke. Studies in vitro and in vivo have demonstrated that I/R injury causes a release of mtDNA into microglial cytoplasm, promoting the polarization of microglia towards the pro-inflammatory M1-like phenotype and restraining anti-inflammatory M2-type microglia polarization through activation of the STING pathway [193]. Accordingly, pharmacologic inhibition of STING with the low molecular weight inhibitor C-176 in mice, subjected to experimental ischemic stroke, reduced I/R-induced brain infarction, edema, neuronal injury/degeneration as well as sensorimotor and cognitive impairments, whereas the intracerebroventricular administration of mtDNA worsened brain tissue damage and functional deficits [193]. Moreover, treatment with C-176 was sufficient to prevent the detrimental effects of exogenous mtDNA on stroke outcomes [193]. Similarly, in a mouse model of ischemic stroke, the elevated occurrence of dsDNA in the cytoplasm of astrocytes and microglia across the penumbra as early as 6 h after onset of cerebral ischemia was shown [215]. However, the origin of cytoplasmic dsDNA is not fully clear. It may leak from dysfunctional mitochondria [298], but uptake of extracellular dsDNA, massively released from necrotic neurons upon I/R injury, might also play a role.

Accordingly, the exposure of in vitro cultured microglia to conditioned medium derived from neurons subjected to OGD caused substantial M1-like polarization, which was, however, attenuated by either addition of the mtDNA inhibitor dideoxycytidine or the knockdown of the key cytosolic dsDNA sensor cGAS, working upstream from STING [174]. It is worth mentioning that neurons and astrocytes can exchange damaged mitochondria with each other for disposal and recycling after stroke raising the possibility that the elevated mtDNA in microglial cytoplasm of ischemic brain may be partly derived from astrocytes and neurons through cell-to-cell communication [67, 152, 193].

Furthermore, experimental evidence was provided that pharmacological blockade of cGAS with the irreversible STING-inhibitor A-151 was sufficient to alleviate cerebral damage and functional defects [215]. The improved stroke recovery upon A-151 treatment was further accompanied by reversal of cGAS/STING-mediated upregulation of AIM2 inflammasome- and pyroptosis-associated molecules, neutrophil infiltration as well as the production of pro-inflammatory factors and pyroptosis in microglia [215]. Consistently, cell-specific genetic ablation of cGAS in microglia protected against brain damage, improved neurobehavioral performance, and reduced cell death after stroke to a similar extent as compared to the pharmacological inhibition of cGAS through A-151 [215].

Of note, circulating neutrophils, the first immune cells to be recruited into the brain tissue after stroke by excessive local release of pro-inflammatory cytokines and DAMPs, may further exert harmful effects by subsequent release of NETs, and NETs themselves directly allow dsDNA to be released into the microenvironment, thus forming a positive feedback loop of inflammation [215]. Indeed, numerous studies have localized NETs in the perivascular space of infarcted lesions in specimens from ischemic stroke patients and corresponding animal models [474]. CfDNA together with histones as major network structure of NETs, was firstly shown to act as a reaction platform for blood cell adhesion, platelet activation and the induction of blood coagulation promoting thrombosis and limiting the fibrinolytic effect of t-PA, the only approved pharmacological therapy for acute ischemic stroke in humans [474]. In fact, plasma levels of cfDNA and NETs (as biomarkers) are significantly increased in patients with acute stroke and are associated with t-PA-resistance as well as with increased disease severity and mortality (Table 3) [80, 210, 460]. Furthermore, treatment of blood clots obtained from ischemic stroke patients with DNase1 ex vivo substantially increased t-PA-induced thrombolysis in comparison to t-PA alone [93, 202, 253]. Moreover, either free or NET-associated extracellular histones provide a strong cytotoxic potential for different cell types that should not be neglected in searching for DNase-based therapeutic measures as mentioned above [327].

In murine ischemic stroke, the degradation of NETs by systemic application of DNase1 reduced BBB breakdown and increased neovascularization and vascular remodeling after stroke to a similar extent as compared to neutrophil depletion by injection of anti-Ly6G antibody or blockade of PAD4, an enzyme essential for NET formation, respectively [184]. PAD4 inhibition also reduced stroke-induced STING-mediated production of IFN-β. Consistently, STING knockdown and IFN receptor-neutralizing antibody treatment decreased BBB breakdown and increased vascular plasticity [184]. In a follow-up study using a mouse model of thrombotic middle cerebral artery occlusion, the same research group demonstrated that t-PA-induced neutrophil recruitment, NET formation, BBB breakdown and cerebral hemorrhage, a most feared clinical complication of t-PA-mediated therapy for acute stroke patients, were effectively alleviated by either DNase1 treatment or PAD4 deficiency [397].

Furthermore, NETs were revealed to be essential for the t-PA-induced upregulation of cGAS/STING and the downstream pro-inflammatory type 1 IFN signaling in microglia and infiltrating macrophages, as DNase1 and ablation of PAD4 substantially reduced the activation of the cGAS/STING pathway and the production of IFN-β and IL-6 in mice subjected to photo-thrombotic stroke and treatment with t-PA [397]. Accordingly, DNase1-mediated reversal of microglia activation, cerebrovascular protection and anti-hemorrhagic effects after ischemic stroke were abolished by co-administration of the cGAS product cGAMP, whereas cGAS deficiency rescued t-PA-associated BBB disruption and cerebral hemorrhage [397]. Beneficial effects of DNase1 treatment alone or in combination with t-PA on the outcome from ischemic and hemorrhagic stroke were confirmed in further preclinical animal studies [70, 80, 297, 361, 419, 444, 460] (detailed information is provided in Table 2).

Conclusively, recent experimental studies suggest that brain-resident microglia and infiltrating neutrophils may interact synergistically to coordinate dsDNA-induced inflammatory responses and culminate in the expansion of ischemic infarction. Thus, inhibitory targeting of NETosis and innate DNA-sensing signaling may be promising therapeutic interventions to treat ischemic stroke [215].

### Multiple sclerosis

#### Etiology and pathogenesis of multiple sclerosis

MS is an inflammatory demyelinating disease of the CNS affecting mainly young people aged between 20 and 40 at disease onset. Initial symptoms are diverse but the most frequent ones are visual disturbances, paresthesias, ataxia and muscle weakness [98]. In 80–85% of patients, the course of the disease is associated with periods of increasing neurological symptoms (relapses) alternating with remissions (relapsing–remitting course of MS, RRMS). With time, for most patients the disease passes into a secondary progressive course (SPMS) characterized by continuous progression of symptoms. In 10–15% of patients the neurological deficit increases continuously from the moment of the disease manifestation (primary progressive form of MS, PPMS) [195]. Although incompletely understood, the etiology of MS presumably involves interaction between genetic, environmental, and other factors triggering an aberrant autoimmune attack resulting in damage to myelin and axons. In the pathogenesis of MS, two mutually complementary processes can be distinguished: the autoimmune neuroinflammation directed against the myelin sheath components that actively develops during the early stages of the disease, and neurodegeneration, which plays a leading role in the progression of MS [195]. At the cellular level, pathological hallmarks include peripheral activation of autoreactive myelin-specific T cells, their migration into the CNS and reactivation of self-reactive T cells by resident and infiltrating activated antigen-presenting cells (APCs), demyelination, remyelination, gliosis, and axonal/neuronal degeneration [98]. In the initial stages of MS development, autoreactive CD4+ T helper type 1 (Th1) and CD4+ T helper type 17 (Th17) cells are elevated in the CNS, where they initiate inflammation and neuronal cell death by producing IFN-γ and IL-17, respectively [439]. In addition, CD4+ regulatory T cells (Tregs), which normally prevent damage to host cells by limiting the immune response, are decreased in the frequency and suppressive function of MS [439].

#### The role of extracellular RNAs during neuroinflammation in multiple sclerosis

Over the past decade, many studies have identified a large set of circulating cell-free or cell-associated ncRNAs that are dysregulated in MS, particularly in a lineage-related manner or in specific cell populations as well as during particular stages/subtypes of MS, providing new MS-specific biomarkers to predict disease activity and progression (summarized in Table 4) or therapy response. Furthermore, some of these regulatory ncRNAs have been functionally characterized to play critical roles in MS pathogenesis (for a comprehensive overview see also [128, 439]).

**Table 4:** Summary of SENAs associated with disease activity and severity of multiple sclerosis in human patients

SENA	Study population	Sample type	RNA/DNA name	Findings	References
miRNA	20 HC 151 MS	CSF	miR-142-3p	Upregulated in MS patients Expression positively correlated with disease progression	[73]
	30 HC 30 MS	PBMC	miR-193a	Downregulated in MS patients Expression inversely correlated with disability level and disease severity	[326]
	32 HC 15 CIS 61 RRMS	Whole blood	miR-146a, miR-155	Upregulated in RRMS compared to HC and CIS Expression positively correlated with disability level	[331]
	30 HC 15 RRMS 11 SPMS 4 PPMS	Serum	miR-146a, miR-155	Upregulated in RRMS and SPMS compared to HC Expression positively correlated with disability level	[337]
	21 HC 24 RRMS	Plasma	miR-125a-5p	Upregulated in RRMS compared to HC Increased in RRMS patients with a higher disability level	[130]
	10 HC 25 RRMS	Blood CD8+ T cells	miR-146a-5p	Downregulated in RRMS compared to HC Increased in RRMS patients with a higher disability level	[97]
			miR-155	Downregulated in RRMS compared to HC Expression positively correlated with disability level and duration of disease	
	20 HC 25 CIS 117 RRMS 24 PMS	CSF	let-7b-5p	Upregulated in RRMS compared to PMS Expression inversely correlated with central and peripheral inflammation in non-PMS (CIS + RRMS) Positively correlated with cognitive performance in non-PMS Negatively correlated with disability level in PMS	[252]
	15 HC 15 RRMS (relapse) 16 RRMS (remission) 16 PPMS 15 SPMS	Serum	miR-572	Downregulated in the overall group of MS patients compared to HC Downregulated in PPMS and RRMS (remission) compared to HC Higher in SPMS compared to PPMS Higher in RRMS (relapse) compared to RRMS (relapse) Expression positively correlated with disability level in the overall group of MS patients	[250]
	28 CIS-CIS 30 CIS-RRMS	CSF	miRNA-181c	Expression level positively correlates with probability of conversion to RRMS after 1 year	[5]
	42 HC 25 RRMS (relapse) 18 RRMS (remission)	Peripheral blood leukocytes	miR-326	Upregulated in relapsing phase of MS patients compared to HC and patients in remitting phase Enhanced expression in the CD4+ T cell population but not in the CD8+ T cell or non–T cell populations of patients with relapsing MS	[91]
	20 HC 20 RRMS (relapse) 20 RRMS (remission)	Peripheral blood lymphocytes	miR-326, miR-26a	Upregulated in relapsing phase of MS patients compared to HC and patients in remitting phase	[157]
	32 HC 50 RRMS 51 SPMS	Plasma	miR-92a-1	Differently expressed in RRMS versus SPMS, and RRMS versus HC Expression associated with disability level and disease duration	[125]
lncRNA	30 HC 30 CIS 30 RRMS (relapse) 30 RRMS (remission) 30 SPMS	Serum	RUNXOR	Downregulated in all MS patients in comparison with HC Within the MS patients’ groups, the downregulation increased with the progression of the disease, with the lowest value observed in the SPMS patients and the highest value in CIS patients In RRMS patients, relapse was associated with lower expression than remission	[148]
	30 HC 30 RRMS (relapse) 30 RRMS (remission)	PBMC	HOTAIR, THRIL, H19	Upregulated in relapsing phase of MS patients compared to HC and patients in remitting phase Expression positively correlated with increased levels of TNF-α and MMP9	[348]
			NKILA	Downregulated in relapsing phase of patients compared to patients in remitting phase and HC Expression negatively correlated with the level of TNF-α	
			ANRIL	Upregulated both in relapsing and remitting phases of MS patients compared to HC Expression higher in patients in remitting phase than those in relapsing phase Expression positively correlated with increased level of IL-6	
	10 HC 20 RRMS	Whole blood	MEG3a	Downregulated in MS patients Expression negatively correlated with disability level	[271]
	43 RRMS (mild) 21 RRMS (severe)	Whole blood	ENSG00000260302, ENSG00000270972, ENSG00000272512, ENSG00000223387	Differentially expressed between mild and severe phenotype MS	[140]
	50 HC 100 MS	Serum	MAGI2-AS3	Downregulated in MS patients Expression inversely correlated with disability level	[194]
	104 HC 108 MS	Serum	GAS5	Upregulated in MS patients Expression positively correlated with disability level	[336]
circRNA	27 HC 18 RRMS (relapse) 27 RRMS (remission)	PBMC	circRNA_101145, circRNA_001896	Downregulated in remitting phase of patients compared to patients in relapsing phase and HC Expression positively correlated with disability level	[274]
	27 HC 19 RRMS (relapse) 28 RRMS (remission)	PBMC	circRNA_101348, circRNA_104361	Upregulated in relapsing phase of patients compared to patients in remitting phase and HC Expression positively correlated with gadolinium enhancement on brain MRI	[488]
cfDNA	64 HC 60 RRMS	Whole blood	mtDNA	Copy number reduced in RRMS patients Copy number inversely correlated with disease duration	[6]
	23 HC 21 MS	CSF	mtDNA	Copy number increased in MS patients Copy number negatively correlated with disease duration	[380]
	23 NINDC 50 RRMS 13 PPMS 27 SPMS	CSF	mtDNA	Copy number increased in PPMS and SPMS compared to NINDC Copy number positively correlated with disability level, T2 lesion volume and brain atrophy	[209]

ANRIL: Antisense non-coding RNA in the INK4 locus; cfDNA: cell-free DNA; circRNA: circular RNA; CIS: clinically isolated syndrome; CSF: cerebrospinal fluid; GAS5: growth arrest-specific 5; H19: H19 imprinted maternally expressed transcript; HC: healthy control; HOTAIR: Hox antisense intergenic RNA; lncRNA: long non-coding RNA; MEG3a: maternally expressed gene 3a; miRNA: microRNA; MRI: magnetic resonance imaging; MS: multiple sclerosis; mtDNA: mitochondrial DNA; NINDC: non-inflammatory neurologic disease controls; NKILA: NF-κB interacting lncRNA; PBMC: peripheral blood mononuclear cells; PMS: progressive MS; PPMS: primary-progressive MS; RRMS: relapsing–remitting MS; RUNXOR: RUNX1 overlapping RNA; SENA: self-extracellular nucleic acid; SPMS: secondary-progressive MS; THRIL: TNF-α and heterogenous nuclear ribonucleoprotein L related immunoregulatory lincRNA

#### MicroRNAs and neuroinflammation in multiple sclerosis

Among them are several miRNAs that influence the differentiation of pro-inflammatory Th1 cells and Th17 cells (e.g. miR-326, miR-448, let-7e), the development of Tregs (miR-106b, miR-25), and the alteration of the Th2 to Th1 response in MS (miR-128, miR-27b, miR-340) [439]. In addition to T cells, several important miRNAs regulating the activation and effector functions of APCs such as B cells (e.g. miR-320a, miR-132), blood-derived macrophages and microglia (e.g. miR-155, miR-124), have been identified to be differentially expressed in patients with MS [439]. Similar to immune cells, aberrant expression of miRNAs in resident CNS cells presumably contributes to the mechanisms underlying inflammation in MS [439]. In active human MS lesions, 20 miRNAs have been identified to be at least twice more abundant and 8 miRNAs at least twice less abundant than in normal white matter. Interestingly, astrocytes contained all 10 miRNA that were most strongly upregulated. Amongst, the local upregulation of the three miRNAs miR-34a, miR-155 and miR-326 is supposed to be linked to the local downregulation of CD47 (considered as a “Don’t eat me” signal) on brain-resident cells and myelin in active MS lesions, thereby unleashing macrophages for tissue destruction [180].

The pro-inflammatory miR-155 is highly expressed in the serum and in CNS lesions of MS patients [365]. Both, global or T cell-specific knockout of miR-155 in mice confers resistance to experimental autoimmune encephalomyelitis (EAE), a mouse model of MS, by reducing the encephalogenic potential of CNS-infiltrating Th17 T cells [365].

A recent study has designated miR-223-3p as a negative regulator of NLRP3 inflammasome engagement in activated macrophages/microglia, both in experimentally-induced demyelination and human MS lesions [122]. Systemic delivery of miR-223-3p mimics to mice following lysolecithin-induced demyelination suppressed NLRP3 inflammasome activity in both macrophages and microglia, and resulted in a significant reduction of axonal injury within demyelinated lesions [122].

Furthermore, miR-409-3p and miR-1896, upregulated in IL-17-activated astrocytes in vitro and in EAE mice in vivo, co-ordinately promoted the production of inflammatory cytokines in reactive astrocytes through the SOCS3/STAT3 pathway and enhanced astrocyte-directed chemotaxis of CD4+ T cells, aggravating demyelination in EAE mice [233].

Overexpression of miR-99a, another anti-inflammatory miRNA, alleviated EAE development by promoting Tregs and inhibiting Th1 cell differentiation through suppression of mechanistic target of rapamycin (mTOR)-regulated glycolysis in CD4+ T cells [137].

The systemic knockdown of pro-inflammatory miR-181c resulted in attenuated EAE clinical symptoms and decreased the spinal cord inflammation and demyelination, along with a decreased Th17 cell population [467]. MiR-181c knockdown rendered T cells less sensitive to TGF-β-induced Smad2/3, enhancing the expression of IL-2, which has been reported to inhibit Th17 cell differentiation [467].

Moreover, overexpression of anti-inflammatory miR-23b resulted in a strong resistance to EAE by inhibiting the migration of pathogenic T cells to the CNS through targeting C-C motif chemokine ligand 7 [464].

Altogether, numerous miRNAs which were found to be dysregulated either in blood-borne immune cells, brain-resident cells or body fluids of MS patients are predicted to regulate immune/inflammatory responses. As a consequence, in-depth in silico studies are needed to identify their target genes and related immune/inflammatory pathways. Further gain- and loss-of-function studies in animal MS models will also be necessary to evaluate the therapeutic potential of the most promising miRNA candidates.

#### Long non-coding RNAs and neuroinflammation in multiple sclerosis

Like miRNAs, lncRNAs play an important role in both innate and acquired immunity. In the last years, a continuously growing number of deregulated lncRNAs have been identified in serum, peripheral blood mononuclear cells (PBMCs) and blood samples of MS patients (summarized in [128, 285]). A higher abundance of three circulating lncRNAs in the serum of patients suffering from RRMS has been reported: NEAT1, RNA component of 7SK nuclear ribonucleoprotein (RN7SK) and TUG1 [329]. These three lncRNAs are involved in specific regulatory functions: NEAT1 promotes expression of the CXC motif chemokine ligand 8 gene encoding IL-8 via relocation of splicing factor proline- and glutamine-rich (SFPQ), RN7SK is involved in regulation of CD4+ T cells, and TUG1 is a component of the p53 regulatory network [285].

Similarly, higher levels of lncRNA growth arrest-specific 5 (GAS5) are found in amoeboid-shaped microglia in MS patients. Functional studies had demonstrated that GAS5 has pro-inflammatory properties as it suppressed microglia M2 polarization through repression of TRF4 transcription by recruiting the polycomb repressive complex 2 (PRC2). Consequently, intracerebroventricular transplantation of GAS5-depleted microglia attenuated disease progression and promoted re-myelination in animal models of MS [355].

Moreover, linc-MAF-4 levels were considerably higher in PBMCs from MS patients than in healthy controls. Linc-MAF-4 exacerbates MS pathogenesis by altering the Th1/Th2 ratio and by targeting musculo-aponeurotic fibrosarcoma (MAF), a Th2 cell transcription factor required for Th2 differentiation [439].

The lncRNA MALAT1 is downregulated in both the CNS of human MS patients and in spinal cords of EAE animals at the peak of disease [258]. The knockdown of MALAT1 in EAE mice exacerbated autoimmune neuroinflammation through changing the pattern of macrophage differentiation towards a M1-like phenotype as well as enhancing T cell differentiation towards pathogenic Th1 and Th17 cells, while impeding the differentiation of protective Treg cells, collectively pointing to a potential anti-inflammatory effect for MALAT1 in the context of MS [258].

Conclusively, several pieces of evidence have demonstrated a promising role of lncRNAs as potential diagnostic and prognostic biomarkers in MS patients. However, studies in this area have just begun, and further research is required to determine the specific molecular mechanisms and biological functions of these lncRNAs in the pathogenesis of MS.

#### Circular RNAs and neuroinflammation in multiple sclerosis

While the contribution of miRNAs and lncRNAs to the progression of MS is well accepted, the role of circRNAs in the pathogenesis of MS is still largely obscure and only a few reports have addressed this topic. More than 400 circRNAs that were differentially expressed in blood samples of RRMS patients have been identified [165]. From these, two circRNAs, circ_0005402 and circ_0035560, have been confirmed to be downregulated in the MS population upon several validation steps. Both of them are located inside the annexin A2 (ANXA2) gene, which had not been previously related to MS but other immune-mediated diseases. Moreover, ANXA2 has also been reported to be a target of miR-155, a critical miRNA in neuroinflammation at the BBB and relevant in Th1 and Th17 cell differentiation and myeloid cell polarization in MS as mentioned above [165]. Based on the fact that ANXA2 and miR-155 are inversely regulated in MS patients, a complex interaction between miRNA, mRNA and circRNAs can be anticipated in the course of MS [165].

Similarly, a recent study on circRNA expression profiles in PBMCs revealed more than 900 transcripts to be differentially expressed between patients with RRMS in relapse and healthy controls, and demonstrated the overexpression of circ_101348, circ_102611, and circ_104361 in MS patients [488]. Bioinformatic analysis revealed 15 miRNAs interacting with these circRNAs in a complementary manner and led to the discovery and validation of three protein-coding RNAs upregulated in patients with RRMS during relapse. Two of these, adenylate kinase 2 and Ikaros family zinc finger protein 3 (IKZF3), have previously been implicated in B cell function [488].

Moreover, circ_0000518 was shown to be upregulated in CSF and in the peripheral blood of MS patients as well as to exacerbate EAE by promoting macrophage/microglial M1 polarization through the fused in sarcoma (FUS)/calcium/calmodulin-dependent protein kinase kinase beta (CaMKKβ)/AMP-activated kinase (AMPK) pathway [173].

A previous study showed an upregulated expression of circINPP4B in Th17 cells from mice with EAE and during Th17 differentiation in vitro [144]. The silencing of circINPP4B inhibited Th17 differentiation and alleviated EAE, characterized by a reduced demyelination and Th17 infiltration in the spinal cord. Mechanistically, circINPP4B served as a sponge that directly targeted miR-30a to regulate Th17 differentiation [144].

Together, emerging recent data clearly support the notion of circRNAs involvement in the pathogenesis of MS. Several lines of evidence indicate that circRNAs may play a distinctive role in both adaptive and innate immune responses in MS by determining the availability of miRNAs for their known post-transcriptional regulation of genes related to immune cell polarization and immune effector functions [487]. In addition, contrary to other RNA species they are very stable in the blood and other biological fluids and thus might be considered as valuable biomarker candidates for MS [487].

#### The emerging roles of extracellular DNA in neuroinflammatory processes during multiple sclerosis

In recent years, impaired mitochondrial function is increasingly recognized as a key pathological hallmark of MS. Demyelination leads to an increase in energy demand in order to maintain an appropriate intra-axonal ion balance and could thereby affect mitochondria at multiple levels [209]. Among others, disturbances in mitochondrial dynamics may promote the release of mitochondrial DAMPs, particularly cf-mtDNA [71]. On entering the cytoplasm or the extracellular space, mtDNA can become pro-inflammatory and initiate innate and adaptive immune responses by activating cell surface and intracellular receptors in both resident and infiltrating cells [134]. In accordance with the possible role of mitochondrial dysfunction in the pathology of MS, increased levels of mexDNA were found in blood plasma and lumbar CSF samples of patients suffering from progressive forms of MS [115, 209, 277] (Table 4). Higher plasma levels of mexDNA were accompanied by increased plasma concentrations of pro-inflammatory cytokines [277]. Moreover, enhanced mexDNA concentrations in the CSF of patients with PMS correlated with high T2 lesion volumes and were inversely related to normal brain volume, indicating that the increased concentration of mexDNA is mostly due to ongoing neuro-axonal damage, which is known to be more extensive in progressive forms of MS [209].

Significantly higher levels of mexDNA were also found in lumbar CSF samples taken from patients with RRMS, which, however, declined over the disease duration [115, 380]. There is increasing evidence that the pathological mechanisms of PMS and RRMS are different. While relapses are thought to be caused by acute focal inflammation, relapse-independent progression is the clinical consequence of more diffuse inflammatory and neurodegenerative processes [150]. Thus, high levels of mexDNA in RRMS might be predominantly due to active release in response to a stimulus, and could reflect early inflammatory activity rather than neuronal loss. Accordingly, RRMS patients treated with Fingolimod, which limits autoreactive inflammation in the CNS by acting on sphingosine-1-phosphate receptors, which are present on peripheral immune cells as well as glial and nerve cells, had significantly lower mexDNA copy levels at follow-up compared to baseline [209]. In contrast, *post-mortem* ventricular CSF analysis revealed a decreased mexDNA abundance and integrity in patients suffering from PMS, which, however, did not correlate with protein markers of neurodegeneration [236].

Despite the potential of mexDNA as a reliable diagnostic and prognostic biomarker for MS (Table 4), only a few studies have addressed the mechanistic link between mtDNA release, inflammatory response, and progression of MS. In this regard, the stimulation of human microglia with mexDNA in vitro increased ROS production, but did not affect antigen presentation properties and expression of pro-inflammatory cytokines [278], implying that increased levels of mexDNA might contribute to the chronic and dysregulated activation of microglia, as demonstrated in MS and other neurodegenerative diseases. In a similar fashion, mtDNA-containing neuron-derived mitochondrial lysates, but not mitochondrial lysates from mtDNA-depleted cells, could activate inflammatory pathways in cultured neuronal and microglial cells [409].

Consistently, injection of mtDNA into mouse hippocampus increased NF-κB signaling, TNF-α expression and astrocyte proliferation [410]. Cultured microglial cells transfected with mtDNA revealed a pro-inflammatory microenvironment by activation of the cGAS/STING pathway as one of the primary aberrant cytoplasmic DNA sensors [221]. Moreover, control or oxidant-initiated degraded mtDNA triggered a pro-inflammatory response in mouse primary astrocytes [260]. Overall, emerging evidence point to mexDNA, and even more when degraded by oxidation, as an important DAMP in MS and other neurodegenerative diseases associated with inflammation and oxidative imbalance.

Neutrophils, as the most abundant circulating and first-responding innate myeloid cells, have been increasingly demonstrated to play crucial roles in the development and pathology of MS, among others, by the formation and release of mtDNA-containing NETs [68]. Circulating NETs were found to be elevated in the serum of RRMS patients compared to healthy controls [275]. NETs were, however, not detected in CSF samples of MS patients, corresponding with previous reports that pointed to the absence of neutrophils within the CNS of MS patients. Yet, it was suggested that cytotoxic components of NETs may contribute to BBB damage in this disease [251, 370]. Accordingly, in mice subjected to EAE the depletion of NET-associated proteins such as myeloperoxidase and neutrophil elastase caused an attenuated disease severity and BBB breakdown [440, 450]. Also, the transmigration of murine neutrophils through an activated cerebrovascular endothelium induced a pro-inflammatory, neurotoxic phenotype that subsequently leads to the release of NETs containing de-condensed DNA associated with proteases [10, 68]. The blockade of histone–DNA complexes attenuated transmigrated neutrophil-induced neuronal death, whereas the inhibition of key neutrophil proteases in the presence of transmigrated neutrophils rescued neuronal viability [10].

Furthermore, upon activation, CD4+ T lymphocytes have been shown to release extracellular oxidized DNA that provides autocrine costimulatory signals to T cells [57]. Pharmacological inhibition of mitochondrial ROS during the priming phase of EAE abolished the extrusion of DNA by CD4+ T cells and reduced T cell priming against myelin. Moreover, mitochondrial ROS blockade during established EAE markedly ameliorated the disease severity, thereby dampening autoimmune inflammation of the CNS [57].

Neuroinflammation is also associated with high levels of extracellular ATP, which is released from activated cells, mostly astrocytes, or leaking from injured or dead cells, to serve as a DAMP that activates pro-inflammatory responses [55]. Neurons, glia and infiltrated immune cells can sense ATP as well as other extracellular nucleotides (e.g., ADP, UTP, and UDP) via specific purinergic P2 receptors [55]. The P2X7 receptor, one of the most abundant P2 receptors in the CNS and activated by ATP, triggers a cascade of responses including the release of pro-inflammatory mediators and excitatory neurotransmitters, induction of cell proliferation but also cell death [55]. In line with the role of P2X7 in MS, the expression of this receptor is significantly elevated in neurons, astrocytes, and microglial cells/macrophages of MS patients and in brain samples from rodents subjected to EAE. Consistently, mice lacking P2X7 are less susceptible to EAE, while EAE is ameliorated by pharmacological blockade of P2X7 signaling [261, 339].

Conclusively, exDNAs and extracellular nucleotides that are accumulating in the brain during MS to promote inflammatory processes, are potential therapeutic targets for MS. Yet, upon MS-related injury conditions it remains to be studied whether direct interactions between polyanionic exRNAs or exDNAs with basic proteins in the myelin sheet might cause autoimmune reactions due to the generation of neo-antigen complexes. Such a pathomechanism has been uncovered for the autoimmune disease heparin-induced thrombocytopenia (HIT) where complexes between extracellular nucleic acids and the basic protein platelet factor 4 induce the formation of HIT-antibodies [167].

### Alzheimer's disease

#### Etiology and pathogenesis of Alzheimer's disease

AD is the most prevalent neurodegenerative disorder related to age, which is clinically associated with a global cognitive decline and progressive loss of memory and reasoning [257]. The defining neuropathological features of AD comprise deposition of extracellular amyloid plaques and intraneuronal neurofibrillary tangles (NFTs), consisting of densely packed amyloid-β (Aβ) peptides, derived from the amyloid precursor protein (APP) via sequential proteolytic cleavage by β- and γ-secretases, and the hyper-phosphorylated microtubule-binding protein tau (tubulin associated unit), respectively [257]. According to the prevailing amyloid cascade hypothesis, the accumulation of these proteins appear to follow a temporal sequence, with Aβ accumulation triggering a cascade of events comprising NFT formation, synaptic and mitochondrial dysfunction, and neuronal loss [72, 257].

Chronic neuroinflammation is also a typical feature of AD pathogenesis. It is widely accepted that microglia-mediated neuroinflammatory responses may promote neurodegeneration in AD [443]. Microglial activation precedes neuronal loss in patients with AD, and recent genome-wide association studies have revealed that microglial genes such as CD33, triggering receptor expressed on myeloid cells 2 (TREM2) and human leukocyte antigen-DR isotype (HLA-DR) are associated with susceptibility to late-onset AD [443]. Aβ oligomers and fibrils are capable of priming microglial cells through interactions with various receptors, which enhance the production of inflammatory cytokines and chemokines, and make microglia more susceptible to secondary stimuli, thereby promoting chronic activation of primed microglia [156, 257]. In addition to microglia, astrocytes undergo complex, brain region- and disease stage-specific changes in the course of AD. Astrocytic atrophy and loss of function, preceding the formation of senile Aβ-plaques, can contribute to early AD pathophysiology, including synaptic dysfunction, impaired synaptogenesis and cognitive deficits [13].

In addition, hypertrophic astrocytes have been described to reside within the vicinity of senile Aβ-plaques, taking part in the proteolytic clearance of Aβ-peptide [257]. However, similar to microglia, astrocytes also sense Aβ-aggregates in a TLR/RAGE-dependent manner, which leads to increased production of neurotoxic factors, including ROS, NO, pro-inflammatory cytokines and chemokines. Excessive production of neurotoxic factors disturbs astrocyte's APP processing homeostasis, which leads to increased Aβ-peptide load and toxicity [343]. Although Aβ-peptide is probably the key inducer of neuroinflammation in AD, it does not exclude the possibility that other intrinsically generated molecules such as SENAs might also contribute.

#### Extracellular RNAs and their neuroinflammatory implications in Alzheimer's disease

Emerging evidence indicates that regulatory ncRNAs such as lncRNAs, miRNAs, and circRNAs exert crucial regulatory effects in the initiation and development of AD. Compared to healthy controls, the levels of certain ncRNAs and their target mRNAs are significantly altered in the CNS, CSF, and blood of patients affected by AD, highlighting circulating ncRNAs as promising biomarkers for early diagnosis and prediction of AD progression (Table 5). Although the mechanisms are still not fully elucidated, recent studies have further revealed that these highly conserved ncRNAs impact in a convergent as well as divergent manner on core pathophysiological processes underlying AD such as neuroinflammation and oxidative stress, aberrant generation of Aβ-peptide, anomalies in the production, cleavage and post-translational marking of tau, impaired clearance of Aβ-peptide and tau, perturbation of axonal organization, disruption of synaptic plasticity, endoplasmic reticulum stress and the unfolded protein response, mitochondrial dysfunction, aberrant induction of cell cycle re-entry, and apoptotic loss of neurons (systematically reviewed in [204, 266, 466]). According to the scope of this review, the role of certain members from different classes of ncRNAs in neuroinflammation during AD is summarized below.

**Table 5:** Summary of circulating SENAs associated with disease state and severity of Alzheimer’s disease in human patients

SENA	Study population	Sample type	RNA/DNA name	Findings	References
miRNA	20 HC 40 AD 40 MCI	Plasma	miR-483-5p	Upregulated in AD and MCI (early stage AD patients) compared to HC, decreased in AD versus MCI Expression positively correlated with cognitive impairment	[325]
	26 sMCI 19 pMCI	Whole blood	miR-146a, miR-181a	Upregulated in patients with MCI who later converted to AD Expression positively correlated with hippocampal atrophy and disconnections in critical white matter brain regions	[15]
	31 HC 30 MCI 25 AD	Plasma	miR-206	Upregulated in AD and MCI compared to HC Expression positively correlated with cognitive decline in MCI patients over a 4-year longitudinal evaluation	[188]
	86 HC 121 AD	Serum	miR-202	Downregulated in AD patients Expression inversely correlated with cognitive impairment	[86]
	32 HC 66 AD	Serum	miR-27a-3p	Downregulated in AD patients Expression negatively correlated with disease severity	[153]
	53 AD	Plasma	miR-342-5p	Expression inversely correlated with cognitive decline over a 2-year follow-up period	[65]
	41 HC 116 AD	Plasma	miR-21-5p, miR-126-3p	Upregulated in AD patients Expression positively correlated with cognitive impairment	[129]
	60 HC 110 AD	Serum	miR-331-3p	Downregulated in AD patients Expression inversely correlated with cognitive impairment and blood levels of pro-inflammatory cytokines	[232]
	30 HC 48 AD	Serum	miR-374b-5p	Downregulated in AD patients Expression negatively correlated with cognitive impairment	[452]
	19 HC 42 AD	Plasma-derived extracellular vesicles	let-7 g-5p, miR126-3p, miR142-3p, miR146a-5p, mir223-3p	Expression negatively correlated with disease severity	[4]
	45 HC 49 AD	Plasma	miR-146a	Expression positively correlated with cognitive impairment	[249]
	120 HC 20 AD	Plasma	miR‐103, miR‐107	Expression inversely correlated with dementia severity	[392]
	23 HC 23 AD	Serum	miR-34a, miR-29b, miR-181	Upregulated in AD patients Expression positively correlated with cognitive impairment	[2]
	33 HC 33 AD	Serum	miR‑4722‑5p, miR‑615‑3p	Upregulated in AD patients Expression positively correlated with cognitive impairment	[235]
	106 HC 117 AD	Serum	miR-128	Upregulated in AD patients Expression positively correlated with cognitive impairment and blood levels of pro-inflammatory cytokines	[457]
	18 HC 27 AD	Serum	miR-501-3p	Downregulated in AD patients Expression negatively correlated with cognitive impairment	[147]
	62 HC 118 AD	Serum CSF	miR-433	Downregulated in AD patients Expression negatively correlated with cognitive impairment	[396]
	93 HC 108 AD	Serum	miR-193a-3p	Downregulated in AD patients Expression negatively correlated with cognitive impairment	[41]
	98 HC 105 AD	Serum	miR-133b	Downregulated in AD patients Expression negatively correlated with cognitive impairment	[430]
	62 HC 84 AD	Serum	miR-223	Downregulated in AD patients Expression negatively correlated with cognitive impairment	[172]
lncRNA	22 HC 22 AD	Whole blood	BDNF-AS	Upregulated in AD patients Expression positively correlated with cognitive impairment	[84]
	90 HC 90 AD	PBMC	lncRNA-17A	Upregulated in AD patients Expression positively correlated with cognitive impairment	[448]
	32 HC 66 AD	Serum CSF	NEAT1	Upregulated in AD patients Expression positively correlated with disease severity	[153]
	30 HC 48 AD	Serum	MAGI2-AS3	Upregulated in AD patients Expression positively correlated with cognitive impairment	[452]
	120 HC 120 AD	Plasma CSF	MALAT1	Downregulated in AD patients Expression inversely correlated with cognitive impairment	[480]
	78 HC 82 AD	Serum	HOTAIR	Upregulated in AD patients Expression positively correlated with cognitive impairment	[239]
	83 HC 108 AD	PBMC	GAS5	Upregulated in AD patients Expression positively correlated with cognitive impairment and hippocampal atrophy	[49]
	36 HC 45 AD	Plasma	BACE1-AS	Upregulated in severely impaired AD patients Expression positively correlated with cognitive impairment	[116]
circRNA	50 HC 50 AD 20 DLB 40 VaD	Whole blood, Plasma	circ_0003391	Downregulated in AD, but not other types of dementia, as compared to HC Expression negatively correlated with cognitive impairment and hippocampal atrophy	[230]
	40 HC 80 AD	CSF	circ_0002945 (circ-AXL), circ_0032253 (circ-GPHN)	Upregulated in AD patients Correlated with elevated AD risk Expression positively correlated with cognitive impairment	[217]
			circ_0030777 (circ-PCCA), circ_0031258 (circ-HAUS4)	Downregulated in AD patients Correlated with decreased AD risk Expression inversely correlated with cognitive impairment	
cfDNA	9 HC 27 AD	Plasma	CNA	Increased concentration in AD patients Concentration positively correlated with cognitive impairment	[290]
	30 HC 30 AD	CSF	8-OHdG (oxidized cfDNA)	Increased concentration in AD patients Concentration positively correlated with duration of illness	[166]
	49 HC 30 spAD 16 rpAD	CSF	mtDNA	Reduced copy number in spAD patients compared to HC and rpAD Low content correlates with the earliest pathological markers of the disease, low Aβ and high p-tau, but not with the marker of neuronal damage t-tau	[303]

8-OHdG: 8-hydroxy-2-deoxyguanosine; AD: Alzheimer’s Disease; BACE1-AS: beta-secretase 1-antisense RNA; BDNF-AS: brain-derived neurotrophic factor-antisense RNA; cfDNA: cell-free DNA; circAXL: circular AXL receptor tyrosine kinase; circGPNH: circular gephyrin; circHAUS4: circular HAUS augmin-like complex subunit 4; circPCCA: circular propionyl-CoA carboxylase subunit alpha; circRNA: circular RNA; CNA: circulating nucleic acid; CSF: cerebrospinal fluid; DLB: dementia with Lewy body; GAS5: growth arrest-specific 5; HC: healthy control; HOTAIR: Hox antisense intergenic RNA; lncRNA: long non-coding RNA; MAGI2-AS3: MAGI2 antisense RNA 3; MALAT1: metastasis associated lung adenocarcinoma transcript 1; MCI: mild cognitive impairment; miRNA: microRNA; mtDNA: mitochondrial DNA; NEAT1: nuclear paraspeckle assembly transcript 1; PBMC: peripheral blood mononuclear cells; pMCI: progressor MCI (subjects who progress to AD); rpAD: rapid progressive AD; SENA: self-extracellular nucleic acid; sMCI: stable MCI (subjects which remain cognitively stable over time); spAD: slow progressive AD; VaD: vascular dementia

#### MicroRNAs and neuroinflammation in Alzheimer's disease

Microglia exposed to pro-inflammatory conditions upregulate miR-155, which increases the production of pro-inflammatory cytokines and reduces the ability of microglia to catabolize fibrillar Aβ_1-42_ in vitro [11, 220]. Similarly, in astrocytes miR-155 is elevated in response to inflammatory stress, and is involved in the upregulation of pro-inflammatory cytokines by targeting SOCS1 mRNA [220]. In a murine AD model, pro-inflammatory miR-155 levels were strongly upregulated and coincided with an increase in microglia and astrocyte activation before the appearance of extracellular Aβ aggregates [138]. The inhibition of miR-155 expression attenuated the upregulation of TNF-α, IL-1β, IL-6, and their receptors, and substantially restored the impaired learning ability of AD rats [227]. Moreover, in neutrophils miR-155 promotes the generation of NETs by increasing the mRNA expression of PAD4 [151].

As another example, miR-146a is abundantly expressed in neurons, microglial cells, and astrocytes, where it acts as a negative feedback regulator of inflammation, whereby miR-146a is upregulated in the temporal cortex of AD patients and hippocampus of AD mice [220, 466]. Here, nasal administration of a miR-146a agomir relieved the progression of AD-associated neuroinflammation by inhibiting the expression of the TLR4 signaling pathway and its related inflammatory genes NF-κB, IL-1 receptor-associated kinase 1 (IRAK1), and TNF receptor-associated factor 6 (TRAF6) as well as reducing the release of inflammatory factors IL-1β, IL-6, and TNF-α [220, 466]. Similarly, microglia-specific miR-146a overexpression in AD mice reduced cognitive deficits in learning and memory, attenuated neuroinflammation, reduced Aβ levels, ameliorated plaque-associated neuritic pathology, and prevented neuronal loss mainly through downregulation of neuroinflammation-related pathways [219]. At the cellular level, anti-inflammatory miR-146a triggered microglial phenotype switching, reduced pro-inflammatory cytokines and enhanced phagocytic function to protect neurons under AD conditions in vitro and in vivo [219]. Autophagy has been proposed as a route of Aβ clearance by microglia that is halted in AD. Accordingly, primary microglia from adult AD mice has been demonstrated to fail to degrade Aβ and expresses low levels of autophagy cargo receptor xext to BRCA1 gene 1 (NBR1), which is required for Aβ proteolysis [100]. Interestingly, NBR1 expression in murine and human AD microglia was negatively correlated with the production of the Mirc1/Mir17-92a cluster member miR-17, which is known to downregulate autophagy proteins. Concordantly, the inhibition of elevated miR-17 in mouse AD microglia improves Aβ degradation, autophagy, and NBR1 expression [100]. Furthermore, in the peripheral blood of AD patients, miRNA-22 levels were found to be negatively correlated with the expression of pro-inflammatory factors [142]. The intracerebroventricular application of miRNA-22-mimic to AD mice inhibited the release of inflammatory cytokines by regulating the inflammatory pyroptosis of glial cells via targeting gasdermin D, and thereby improved the cognitive abilities [142]. In the hippocampus of AD mice the expression of miR-216a-5p was reduced, and the restoration of miR-216a-5p expression improved learning-memory ability and attenuated the inflammatory response of AD mice through targeted inhibition of the HMGB1/NF-κB pathway [338].

There are many other dysregulated miRNAs, which may be involved in AD-related neuroinflammation (for a comprehensive overview see also [220, 234]). However, further gain- and loss-of-function studies are needed to fully decipher the miRNA network in AD-associated neuroinflammation.

#### Long non-coding RNAs and neuroinflammation in Alzheimer's disease

In serum samples of AD patients, the levels of the lncRNA MAGI2-AS3 and its target miR-374b-5p were negatively correlated with disease severity [452]. Similarly, microglial cells exposed to Aβ-peptide in vitro showed elevated MAGI2-AS3 and reduced miR-374b-5p expression. Moreover, overexpression of miR-374b-5p or MAGI2-AS3 knockdown prevented the Aβ-induced upregulation of pro-inflammatory cytokines in microglia [452].

In a rat AD model, hippocampal lncRNA MEG3 levels were substantially reduced. In contrast, MEG3 overexpression improved cognitive impairment, alleviated neuronal damage, as well as reduced the proportion of pro-inflammatory astrocytes and inflammatory cytokine expression [435]. Moreover, lncRNA 4344 overexpression enhanced the expression of the NLRP3-inflammasome and its downstream genes caspase-1, IL-1β, and IL-18 in LPS-treated microglia in vitro, whereas lncRNA 4344 silencing attenuated the inflammatory response. Structural prediction analysis revealed that pro-inflammatory lncRNA 4344 mediates NLRP3 upregulation by negatively targeting miR-138-5p [107]. Detrimental impact of the lncRNA-4344/miR-138-5p/NLRP3 axis on neuronal viability, cognitive function and neuroinflammatory processes was also confirmed in LPS-treated rats in vivo [107].

In cell AD models, the overexpression of the lncRNA MALAT1 reduced IL-6 and TNF-α levels, and increased IL-10 level, while MALAT1 knockdown had the opposite effect. Additionally, MALAT1 reversely regulated miR-125b expression, and rescue experiments revealed that miR-125b prevented the anti-inflammatory effects due to MALAT1 overexpression in Aβ-treated neurons [246]. Similar to the negative correlation of MALAT1 and miR-125b levels determined in Aβ-treated neurons, the abundance of anti-inflammatory MALAT1 in CSF and plasma of AD patients was reduced, whereas miR-125b was increased as compared to healthy controls [246, 480].

Overall, numerous lncRNAs have been identified to be dysregulated in brain and body fluids of AD patients, or have been shown to play crucial roles in neuroinflammation and other processes related to AD pathogenesis in animal disease models. However, more research is required to further elucidate the functions of lncRNAs at molecular and cellular levels, and investigate the full potential of lncRNAs as diagnostic and therapeutic targets in AD.

#### Circular RNAs and neuroinflammation in Alzheimer's disease

In senescent astrocytes, circNF1-419 levels were enhanced and circNF1-419 overexpression promoted autophagy in astrocytes in vitro [83]. In a mouse AD model, circNF1-419 overexpression enhanced autophagy by binding dynamin-1 and adaptor-related protein complex 2 subunit β1 (AP2B1) protein, which was associated with a reduction of AD- and aging-related marker proteins as well as a decrease in the expression of pro-inflammatory mediators [83].

CircHDAC9 levels were lowered in both serum samples of AD patients or brains of AD mice [240]. As demonstrated in an animal model of AD, circHDAC9 acts as a miR-138 sponge, decreasing miR-138 expression, and thus reversing the Sirt1 suppression and excessive Aβ production induced by miR-138 in neurons [240]. Furthermore, circHDAC9 overexpression in neurons in vitro alleviated Aβ-induced pro-inflammatory response and apoptosis through miR-142-5p sequestration [458].

A previous study has shown that ciRS-7, which is downregulated in the brain of AD patients [469], attenuates generation of Aβ-peptide in neurons by promoting the degradation of APP and β-site APP cleaving enzyme-1 (BACE1) protein in a NF-κB-dependent manner [340]. CiRS-7 inhibits the translation of NF-κB and induces its cytoplasmic localization, thus de-repressing the expression of ubiquitin C-terminal hydrolase L1 (UCHL1), which promotes APP and BACE1 degradation [340].

In both, AD mice and Aβ-treated neurons in vitro the expression of circLPAR1 was elevated. The knockdown of circLPAR1 protected cells against Aβ-caused inflammation, oxidative stress, and neuronal apoptosis as well as improved AD-related pathological traits and ameliorated cognitive dysfunctions in vivo [422]. Mechanistically, circLPAR1 inhibits the Sirt1/Nrf-2/HO-1 axis through repression of growth differentiation factor 15 (GDF-15) [422].

As described above, growing evidence demonstrates that circRNAs have been implicated in the pathogenesis of AD. Further efforts are needed to uncover the regulatory roles of circRNAs and their contribution in the underlying mechanisms of AD pathology.

#### The impact of extracellular DNA on neuroinflammation in Alzheimer's disease

As shown in AD animal models and human *post-mortem* brains, accumulation of mutant APP and APP-derived fragments drives mitochondrial dysfunction and mitophagy failure in neurons [205, 316, 377]. An altered concentration of mexDNA in the CSF of AD patients compared with healthy control subjects was found, indicating that CSF mtDNA levels could serve as a biomarker of mitochondrial dysfunction in the etiology of AD and other neurodegenerative disorders [43, 303]. Neuronal and microglial cells exposed to neuron-derived mitochondrial lysates exhibited not only an increased inflammatory gene expression but also showed elevated mRNA and protein levels of APP, while mtDNA-depleted lysates failed to activate inflammatory pathways [409]. Yet, further experimental studies in animal models of AD are required to elucidate whether and how mexDNA contributes to the neuroinflammatory processes in AD.

NETosis is a pathological hallmark of various neurological diseases as already mentioned, and neutrophils together with NETs have also been identified in both human *post-mortem* brain tissue of AD patients as well as in murine models of AD [347, 443]. It is hypothesized that neutrophils migrate inside the parenchyma in areas with Aβ-plaques, where among others Aβ-peptide triggers the formation and release of NETs [302]. Neutrophil depletion or the inhibition of neutrophil intravascular adhesion in mouse AD models improved cognitive decline and neuroinflammation without interfering with the accumulation of Aβ-plaques [31, 61, 385, 443].

The function of exDNA extruded from neutrophils in AD remains unknown, nevertheless, the systemic application of DNase in animal models of stroke and TBI (summarized in Table 2) appears to be effective as an antagonistic treatment. Yet, further studies need to explore the relationship between NET-associated DNA and AD pathogenesis. Further evidence pointing to a therapeutic potential of DNase for treatment of AD comes from a previous clinical case report. A patient with severe dementia and behavioral disturbance secondary to late-onset AD was given 40 mg of recombinant human DNase1 (1500 KU/mg) three times a day in conjunction with continued Memantine therapy (10 mg daily), and apparently produced a rapid and lasting improvement of cognition [346, 363].

### Parkinson’s disease

#### Etiology and pathogenesis of Parkinson’s disease

PD is the second most common neurodegenerative disorder after AD, affecting approximately 2% of the global population over the age of 65 years [14]. Currently, there are two known PD variants: idiopathic or sporadic and rare familial PD. Risk factors of idiopathic PD, most common in late-onset PD cases, include a combination of genetic (e.g. mutations in leucine rich repeat kinase 2” (LRRK2) or glucosylceramidase-β) and environmental factors (e.g. pesticide exposure, prior head injury, rural living, and intensive use of β-blockers). Early-onset PD is often associated with familial inheritance caused by gene mutations in 18 specific chromosomal regions/PD-related loci (i.e. PARK1-18), such as the SNCA gene (α-synuclein; PARK1 and 4), Parkin (ubiquitin protein ligase; PARK2), DJ-1 (PARK7) or LRRK2 (PARK8) [16].

The PD pathology includes two hallmarks: the progressive degeneration of dopaminergic neurons in the *substantia nigra pars compacta* and the formation of Lewy bodies, which largely consist of misfolded and fibrillary forms of α-synuclein in surviving neurons [470]. The main symptoms of PD patients involve bradykinesia, rigidity and resting tremor, whereas non-motor manifestations, such as dementia, depression and dysautonomia are also an integral part of the clinical phenotype [14]. So far, there is no efficient strategy for therapy of the disease, albeit the current dopamine replacement strategies and surgical interventions can provide symptom relief, but still fail to prevent or reverse the underlying pathology [475].

Multiple lines of evidence indicate that neuroinflammatory processes also contribute to PD progression. In particular, pro-inflammatory cytokines are elevated in serum and CSF from patients with PD [29]. From animal models of PD-like neurodegeneration we know that due to the continuous release and accumulation of misfolded α-synuclein, microglial cells are in a chronic or prolonged activation state that substantially contributes to the death of dopaminergic neurons in the midbrain via overproduction of pro-inflammatory cytokines and ROS [108, 470]. Microgliosis has also been demonstrated in the human brain by PD *post-mortem* studies and in vivo PET imaging analysis of diagnosed PD patients [108].

#### The role of extracellular RNAs in the neuroinflammatory cascade during Parkinson’s disease

Numerous studies have proclaimed the impact of regulatory ncRNAs in PD, which may help developing new ways to treat PD. Moreover, circulating ncRNAs have been identified as robust non-invasive prognostic and predictive biomarkers in human PD patients (summarized in Table 6). In line with the focus of the present review on the neuroinflammatory response during CNS diseases, we will introduce several examples out of the families of miRNAs, lncRNAs and circRNAs, highlighting the importance of regulatory ncRNAs for inflammatory processes in the course of PD.

**Table 6:** Summary of circulating SENAs associated with disease state and severity of Parkinson’s disease in human patients

SENA	Study population	Sample type	RNA/DNA name	Findings	References
miRNA	73 HC 75 PD	Plasma	miR-153	Downregulated in PD patients compared to HC Expression positively correlates with disease duration and severity	[415]
	78 HC 78 PD	PBMC	miR- 34a, miR-125a	Reduced in PD patients Expression inversely correlates with disease severity	[429]
	50 HC 68 PD	Serum	miR-374a-5p	Decreased in PD patients Expression is negatively correlated with disease severity	[176]
	126 HC 148 PD	Serum	miR-30c-5p, miR-373	Upregulated in PD patients Expression positively correlates with disease severity	[454]
	44 HC 82 PD	Serum	miR-132-3p, miR-146a-5p	Decreased in PD patients Expression negatively correlates with disease severity	[341]
	14 HC 15 PD	Plasma-derived pure small extracellular vesicles	miR‑34a‑5p	Downregulated in PD patients Expression inversely correlates with disease duration and severity	[136]
	14 HC 30 PD	PBMC	miR-27a-3p	Decreased in PD patients (all stages) Expression negatively correlates with disease severity	[104]
			miR-27b-3p	Increased in PD patients (early stage) Expression decreased along with the disease severity	
	24 HC 23 PD	Serum	miR-214	Upregulated in early stage PD patients Expression negatively correlates with disease duration, severity of anxiety and non-motor symptoms	[426]
	60 HC 80 PD	Serum	miR-150	Reduced in PD patients Expression inversely correlates with blood levels of pro-inflammatory cytokines	[212]
	25 HC 33 PD	PBMC	miR-376a	Increased in PD patients Expression positively correlates with disease severity	[17]
	16 HC 36 PD	PBMC	miR-885, miR-17	Upregulated in PD patients Expression positively correlates with disease severity	[24]
			miR-361	Downregulated in PD patients Expression positively correlates with disease severity	
	222 HC 269 PD	Plasma	miR-132	Increased in PD patients Expression is positively related with the disease duration and severity	[433]
	43 HC 37 PD	PBMC	miR-155-5p	Elevated in PD patients Downregulation by L-dopa treatment	[38]
	80 HC 80 PD	Serum	miR-29a, miR-29c	Decreased in PD patients Expression negatively correlates with disease severity	[18]
	42 HC 44 PD	CSF	miR-144-5p, miR-200a-3p, miR-542-3p	Upregulated in PD patients Expression is positively associated with disease severity	[270]
	112 HC 138 PD	Serum	miR-221	Reduced in PD patients Expression positively correlates with disease severity	[247]
lncRNA	84 HC 97 PD	Serum	TUG1	Upregulated in PD patients compared to HC Expression positively correlates with disease severity and blood levels of pro-inflammatory cytokines	[54]
	13 HC 32 PD	Plasma-derived exosomes	MKRN2–42:1	Decreased in PD patients Expression is positively associated with disease severity	[395]
	196 HC 228 PD	Serum	MALAT1	Increased in PD patients Expression positively correlates with cognitive impairment, motor symptoms severity and blood levels of pro-inflammatory cytokines	[428]
	78 HC 78 PD	PBMC	ANRIL	Elevated in PD patients Expression positively correlates with disease severity	[429]
	30 HC 30 PD	Plasma	MEG3	Decreased in PD patients Expression negatively correlates with disease stage, severity of non-motor symptoms and cognitive decline	[314]
	85 HC 93 PD	Neuronal-derived plasma exosomes	linc-POU3F3	Upregulated in PD patients Expression positively correlates with disease stage, motor symptoms severity and cognitive impairment	[484]
	93 HC 99 PD	Serum	RMST	Increased in PD patients Expression positively correlates with disease severity and blood levels of pro-inflammatory cytokines	[47]
circRNA	100 HC 300 PD	Plasma	circ_0085869, circ_0004381, circ_0017204, circ_0090668	Upregulated in PD patients (all stages) compared to HC Increased in early PD (stage 1) patients compared to HC Elevated levels in late PD (stage 2–5) compared to early PD (stage 1) patients	[472]
	40 PD	Plasma	circEPS15	Expression inversely correlates with motor symptoms severity	[476]
	6 HC 6 PD	Whole blood-derived exosomes	circ_0001535 circ_0000437	Elevated in PD patients Reduced in PD patients after 2-week multidisciplinary rehabilitation	[92]
cfDNA	20 HC 20 PD	CSF	8-OHdG (oxidized cfDNA)	Increased concentration in PD patients Concentration positively correlated with duration of disease	[166]
	10 HC 56 PD	CSF	mtDNA	Reduced copy number in PD patients compared to HC No correlation with cognitive impairment	[310]
	262 HC 363 PD	Peripheral white blood cells	mtDNA	Decreased copy number in PD patients No correlation with cognitive impairment	[309]
	12 HC 21 PD	CSF	mtDNA	Reduced copy number in PD patients compared to HC Higher proportion of mtDNA molecules with deletions in PD patients	[308]

8-OHdG: 8-hydroxy-2-deoxyguanosine; ANRIL: antisense non-coding RNA in the INK locus; cfDNA: cell-free DNA; circRNA, circular RNA; CSF: cerebrospinal fluid; HC: healthy control; linc-POU3F3: long intergenic noncoding RNA POU class 3 homeobox 3; lncRNAs: long non-coding RNA; MALAT1: metastasis associated lung adenocarcinoma transcript 1; MEG3: maternally expressed 3; miRNA: microRNA; MKRN2: makorin ring finger protein 2; mtDNA: mitochondrial DNA; PBMC: peripheral blood mononuclear cells; PD: Parkinson’s Disease; RMST: rhabdomyosarcoma 2-associated transcript; SENA: self-extracellular nucleic acid; TUG1: taurine-upregulated gene 1

#### MicroRNAs and neuroinflammation in Parkinson’s disease

Accumulating evidence demonstrates that aberrant expression of numerous miRNAs might be linked to PD pathogenesis (extensively reviewed in [22, 89, 432]). A total of 125 different miRNAs were significantly altered in the *post-mortem* analysis of the prefrontal cortex from PD patients compared to inconspicuous controls [284]. Among them, various miRNAs are known to modulate the expression of α-synuclein (miR-7, miR-153 and miR-203a-3p) and further PD-causing genes such as Parkin (miR-103a-3p, miR-146a, miR-181a and miR-218), LRRK2 (miR-205 and miR-599), DJ-1 (miR-494 and miR-4639) or PTEN-induced kinase 1 (PINK1) (miR-27a/b). Other miRNAs such as miR-34b/c, miR-126, miR-128, miR-200a, miR-216a, miR-221 or miR-326 are related to the survival and maintenance of midbrain dopaminergic neurons or alternatively affect the α-synuclein-induced neuroinflammation (miR-29c, miR-124, miR-135b, miR-155) [284]. Moreover, a previous study investigated the profile of a selected set of inflammatory miRNAs in the serum of idiopathic PD patients and patients carrying a mutation in the LRRK2 gene [287]. While miR-146a, miR-335-3p, and miR-335-5p supposed to have anti-inflammatory properties, were downregulated in idiopathic PD and LRRK2-PD patients as compared to control cohorts, pro-inflammatory miR-155 was upregulated in LRRK2 but not in idiopathic PD patients [287].

MiR-155, a key regulator of the mammalian immune system, induces neuroinflammation predominantly through the inhibition of endogenous anti-inflammatory molecules such as SOCS1, a negative regulator of pro-inflammatory cytokines, SH2 domain-containing inositol 5´-phosphatase 1 (SHIP1), a negative regulator of TNF-α, or IL-13 receptor-α1 [483]. Moreover, upregulation of miR-155 is believed to be crucial for the conversion of microglia from a quiescent state to a pro-inflammatory M1-like phenotype in the presence of strong inflammatory stimuli [483]. In a mouse model with AAV-mediated overexpression of α-synuclein in the *substantia nigra pars compacta*, miR-155 was found to be upregulated [364], whereas global genetic ablation of miR-155 reduced pro-inflammatory responses to α-synuclein and blocked α-synuclein-induced neurodegeneration. Moreover, miR-155-deficient microglia exhibited a markedly reduced inflammatory response to α-synuclein fibrils, whereas treatment with a synthetic mimic of miR-155 restored the inflammatory response [364].

A recent study demonstrated that miR-485-3p is upregulated in the serum of PD patients and LPS-treated microglia cells in vitro [223]. The release of pro-inflammatory cytokines by activated microglia was even higher in the presence of miR-485-3p mimic, while it was robustly declined after silencing miR-485-3p [223].

Furthermore, intracerebroventricular application of a miR-3473b antagomir to mice treated with 1-methyl-1-4-phenyl-1,2,3,6-tetrahydripyridine hydrochloride (MPTP) promoted autophagy and inhibited the expression of pro-inflammatory factors in microglia within the *substantia nigra pars compacta* [243]. Mechanistically, miR-3473b has been shown to prevent microglial autophagy by targeting TREM2/UNC51-like kinase-1 (ULK1) expression [243].

As an example for anti-inflammatory miRNA in the context of PD, miR-335 has been shown to be downregulated in LPS-treated or LRRK2-overexpressing microglia, in the MPTP-induced PD mouse model as well as in sera from patients with idiopathic PD and those harboring mutations in LRRK2 [288]. In microglia, miR-335 overexpression strongly counteracted the expression of pro-inflammatory genes triggered by either LPS or LRRK2 overexpression [288].

As a further example, miR-let-7a overexpression via injection of miR-7 mimics into the striatum of α-synuclein-induced PD mice suppressed microglia activation and reduced pro-inflammatory cytokine production, which were accompanied by relieved movement disorder and improved spatial memory deficits [451]. Mechanistic investigations revealed that miR-let-7a suppresses the α-synuclein-induced microglial inflammatory response through targeting STAT3 [451].

Similarly, overexpression of miR‐190 in an MPTP‐induced PD mouse model alleviated neuronal damage and inhibited inflammation via negatively regulating the expression and activation of NLRP3 in microglia [356].

These examples imply that deregulated miRNAs in biological fluids do not only represent reliable non-invasive biomarkers for PD diagnosis, prognosis, and treatment response, but could also serve as potential therapeutic targets for the regulation of neuroinflammation and neurodegeneration in PD.

#### Long non-coding RNAs and neuroinflammation in Parkinson’s disease

It is estimated that about 40% of lncRNAs are specifically expressed in brain tissue, and their number far exceeds that of protein-coding genes [475]. Aberrant expression of various lncRNAs has been determined in *post-mortem* brain specimens and biological fluids from PD patients (reviewed in [359, 432]). Compared to non-affected brain tissue, significant changes in the expression of 87 different lncRNAs were identified in the *substantia nigra* of PD patients, among which the significantly upregulated lncRNA AL049437 likely contributes to the risk of PD, whereas the dramatically downregulated lncRNA AK021630 probably leads to the inhibition of PD development [283]. In PBMCs of PD patients 13 lncRNAs exhibited differential expression as compared to healthy controls [351]. Moreover, animal and cell models of PD provide accumulating evidence that lncRNAs contribute to the following pathological processes that ultimately account for the pathological manifestations and clinical symptoms of PD: (i) protein misfolding and aggregation (e.g. SNHG1, long intergenic noncoding RNA-p21 (lincRNA-p21), HOX transcript antisense RNA (HOTAIR)), (ii) mitochondrial dysfunction, oxidative stress, autophagy and apoptosis (e.g. H19, NEAT1, HAGLR opposite strand (HAGLROS), MALAT1), and (iii) neuroinflammation (involving e.g. GAS5). For a more detailed description of the impact of lncRNAs on the pathogenesis of PD the reader is referred to several excellent reviews [242, 245, 420, 475].

With regard to neuroinflammatory processes, GAS5 appears to trigger a pro-inflammatory response of microglia through upregulation of NLRP3 expression via competitively sponging miR-223-3p [423]. Furthermore, lncRNA-p21 sponges miR-181 to promote the activation of microglia and exacerbate neuroinflammation and disease progression by upregulating the expression of protein kinase C-δ in PD models [434]. Gain- and loss-of function approaches in animal and cell models of PD further revealed that lncRNA SNHG1 contributes to neuroinflammation during PD by modulating the miR-7/NLRP3 pathway in microglia [39].

Albeit apparently under-represented, there are few studies on lncRNAs which exhibit remarkable anti-inflammatory properties in the context of PD. For example, overexpression of the lncRNA ZNFX1 antisense RNA 1 (ZFAS1) in 1-methyl-4-phenylpyridinium (MPP+)-treated neurons was demonstrated to prevent NLPR3 inflammasome activation by blocking the miR590-3p-mediated repression of E3 Ubiquitin ligase Mindbomb1 (MIB1)-triggered TXNIP ubiquitination [479].

During the past few years, our understanding of the role of lncRNAs in the development and progression of PD has made substantial progress. However, many lncRNAs are not yet functionally characterized, or we have only minimal information regarding their molecular mechanism of action. Thus, efforts are still needed to identify and functionally characterize lncRNA species involved in the complex pathogenesis of PD. Based on preclinical studies, the use of antisense oligonucleotide-based therapeutics for specific targeting of disease-promoting lncRNAs and their direct delivery across the BBB might represent a powerful and promising approach for PD treatment [371].

#### Circular RNAs and neuroinflammation in Parkinson’s disease

A growing number of studies indicate that circRNAs are implicated in neurological and cardiovascular diseases [197]. In *post-mortem* tissue samples from the *substantia nigra* of individuals without any signs of neuropathology, circRNAs have been shown to accumulate in an age-dependent manner, whereas in the *substantia nigra* of individuals with PD, this correlation is lost and the total number of circRNAs is reduced [145]. Interestingly, an opposite trend was observed in the amygdala and medial temporal gyrus of PD patients [145]. In peripheral blood of PD patients 139 differentially expressed circRNAs were identified. Of them, 78 circRNAs were upregulated, whereas 61 were downregulated [417]. A total of 10 candidate circRNAs (five upregulated and five downregulated) were retrieved for further verification in a larger cohort. Of these circRNAs, circ_103730, circ_101275, and circ_038416 were upregulated, while circ_102850 was downregulated in patients with PD when compared to healthy controls [417]. Similarly, a remarkably differently expressed circRNA landscape was found in plasma samples of PD patients and healthy controls [472]. Further validation revealed a high diagnostic ability of circ_0004381 and circ_0017204 in predicting the early stage of PD from healthy controls, while circ_0085869, circ_0004381, circ_0017204, and circ_0090668 also presented a high ability to distinguish the late stage of PD from early stage [472].

In cell and animal models of PD various circRNAs have been recently demonstrated to be involved in a set of PD's pathogenic processes (extensively reviewed in [88, 90, 456]). Just to give some examples, ciRS-7 and circSNCA have been shown to positively regulate SCNA expression through sponging miR-7 [88]. CircDLGAP4 exerts neuroprotective effects in PD models by preventing the inhibitory effect of miR-134-5p on the cAMP response element-binding (CREB) protein signaling pathway. Circzip-2 exerts modulatory effects on PD progression through targeting miR-60-3p, in a way that miR-60-3p could be upregulated in the absence of circzip-2, and thus, its target mRNAs, coding for PD-protective genes, become downregulated [88]. The circSAMD4A/miR-29c-3p axis modulates autophagy and apoptotic death of dopaminergic neurons through the AMPK/mTOR pathway [88]. CircSLC8A1, upregulated in the *substantia nigra* of PD individuals probably due to oxidative stress, regulates the activity of miR-128, indirectly leading to the deregulation of PD-associated miR-128 targets such as the neurodegeneration and aging-related B lymphoma Mo-MLV insertion region 1 homolog (BMI1), Sirt1, or axis inhibition protein 1 (AXIN1) transcripts [88, 145].

Several circRNAs have also been implicated in neuroinflammation but not in the precise context of PD as yet. For example, circHIPK2 acts as an endogenous sponge for miR-124, and blockage of circHIPK2 inhibited methamphetamine-induced astrocytic activation by lowering the expression of sigma non-opioid intracellular receptor 1 (SIGMAR1) [90]. Moreover, OGD-activated microglia-induced neuronal apoptosis is mediated by circPTK2 that sequesters miR-29b in microglia [90]. Forthcoming studies need to elucidate the circRNA-modulating neuroinflammatory processes during PD pathogenesis as well.

Taken together, circRNAs are differentially expressed in the brain of patients with PD, and growing evidence suggests that they regulate pathological processes in PD. However, it is worth noting that research in this field is still in the initial stage. More insights into the interplay of circRNAs with other regulatory networks involving ncRNAs and proteins may further improve our understanding of PD pathogenesis and could provide a valuable basis for the development of new diagnostic and therapeutic regimes.

#### Extracellular DNA and its impact on neuroinflammation during Parkinson’s disease

Recent studies have linked the amount of mexDNA to neurodegeneration in patients affected by PD. In detail, a low concentration of mexDNA was reported in the CSF of early-stage idiopathic PD patients, suggesting that reduced CSF mexDNA could serve as a biomarker for the onset of PD [123, 310]. The occurrence of reduced amounts of mexDNA may appear contradictory when considering the occurrence of cell death, which is expected to massively release mtDNA. This would elevate rather than depress mexDNA levels [123]. However, following early mitochondrial loss, it is likely that a suppression of baseline release of mtDNA occurs in a compensatory manner. This would explain why only low mexDNA levels, which may anticipate the occurrence of neuronal death, are detected at early stages of neurodegeneration [123]. Accordingly, in dopaminergic *substantia nigra* neurons of healthy individuals, the mtDNA copy number was demonstrated to increase with age in order to maintain the pool of wild-type mtDNA population in spite of accumulating deletions. Such an upregulation is not observed in idiopathic PD patients, resulting in depletion of wild-type mtDNA and an increase in mtDNA deletions [85, 123, 308].

PARK2, encoding the E3 ubiquitin ligase Parkin, is the second most common gene mutated in cases of early-onset familial PD. Upon recruitment and activation by PINK1, Parkin promotes the removal of dysfunctional mitochondria by mitophagy, a selective form of macro-autophagy [301]. Accordingly, in a mouse model that results in the accumulation of dysfunctional mitochondria due to an accelerated generation of mtDNA mutations, the loss of Parkin caused dopaminergic neurodegeneration and motor defects, indicating that the inability to remove mutated mtDNA via mitophagy might result in a PD-like pathology [281, 301]. Moreover, in the absence of Parkin, mtDNA mutational stress results in elevated circulating mtDNA levels, the activation of the DNA-sensing cGAS/STING pathway and an inflammatory phenotype. Remarkably, the genetic inactivation of STING prevented inflammation, motor defects and neurodegeneration in Parkin-deficient mice that had been subjected to mtDNA mutational stress, indicating a connection between mtDNA-induced inflammation and PD [281, 345].

In accordance with the murine model, patients with biallelic or heterozygous PARK2/PINK1 mutations exhibited elevated serum levels of mexDNA and IL-6 as compared to either healthy control subjects or idiopathic PD patients, indicating that mexDNA levels provide a good predictive potential to discriminate between idiopathic PD and PD linked to heterozygous PARK2/PINK1 mutations [29]. Consistently, induced pluripotent stem cell-derived midbrain neurons from PD patients with PARK2 mutations showed deficits in the mitochondrial biogenesis pathway, resulting in mtDNA dyshomeostasis potentially through downregulation of the energy sensor Sirt1, which controls mitochondrial biogenesis and clearance [402]. Moreover, mtDNA dyshomeostasis has been confirmed in *post-mortem* midbrain with PARK2 mutations and was accompanied by an upregulation of microglia overexpressing pro-inflammatory cytokines [402]. Accordingly, parkin-deficient neuron-microglia co-cultures elicited an enhanced immune response when exposed to mtDNA/LPS [402].

Contrary to a previous study demonstrating decreased mexDNA levels in CSF of early-stage patients with idiopathic PD [310], differences in serum mexDNA levels of idiopathic PD patients in an advanced disease state compared to healthy control subjects were not observed [29]. This discrepancy may be explained by the fact that mtDNA levels differ over the course of the disease and vary between tissues. Furthermore, the idiopathic PD group exhibited elevated serum IL-6 concentrations despite comparable mexDNA levels, thus implicating that additional molecular mechanisms independent of the mtDNA-cGAS/STING pathway, may contribute to neuroinflammation during PD progression [29]. Otherwise, the accumulation of oxidative modifications and somatic mutations of mtDNA during the progression of mitochondrial dysfunction might constitute additional parameters that would modulate cGAS/STING signaling and, in turn, IL-6 levels in idiopathic PD patients [29, 281].

Moreover, in addition to cGAS/STING, mtDNA activates other immune receptors as well. Previous studies demonstrated that, in addition to TLR9, newly synthesized oxidized mtDNA can activate the NLRP3 inflammasome [295, 473], whereas double-stranded mtRNA stimulates the RNA-sensing immune receptor MDA5 [82].

Overall, the available experimental evidence points to a close relationship between mitochondrial dysfunction, mtDNA release, inflammatory signaling and loss of *substantia nigra* dopaminergic neurons in PD. If true, then finding ways to reduce the release and accumulation of mtDNA into the cytoplasm or the circulation may be of therapeutic value for PD.

## Perspectives

Based on the here presented translational approaches, several therapeutical regimens have been introduced: For example, specific oligonucleotide-based anti-miRNAs, known as antagomirs, have been designed and successfully used to silence endogenous miRNAs in this regard with potential benefit for therapeutic applications. A recent proof-of-principle study in healthy volunteers revealed that, following a single dose of anti-miR-92a, it efficiently inhibits miR-92a and de-represses its targets in a cell type-specific manner in the peripheral blood compartment as evidenced by single-cell RNA sequencing [1]. It is hoped that such approaches will also be available to target brain-specific miRNAs mentioned in the article, responsible for certain neuroinflammatory/neurodegenerative diseases.

Other types of exRNA such as rexRNA are less well characterized in their structure/function profiles, and considerable effort is needed to decipher the pathophysiological consequences of their appearance in the body. Although the patho-mechanistic insights for rexRNA in several inflammatory-based diseases have been acquired from in vitro and preclinical animal studies, these may not always be transferable to the human disease situation. In addition, as proposed by our group in several experimental studies, the tissue-specific as well as disseminated inflammatory potential of rexRNA can be limited or even inhibited by the application of RNase1, the natural antagonistic endonuclease, present in the circulation as well. Here, recent developments of an IgG1-Fc-fusion protein with RNase1, designated RSLV-132, proved its availability in phase-II studies of inflammatory diseases such as Sjögren's syndrome [35, 304]. Future studies will tell whether RNase1-based therapies may allow to tackle other cases of chronic neuroinflammation, disseminated intravascular coagulation, or even long-Covid syndrome as a scenario of “runaway inflammation”.

Another challenge for improved diagnostic as well as therapeutic measures with respect to exRNAs is related to the (patho-) physiological interplay between different organ systems, characterized by the brain–heart or brain-lung axis, thereby harboring disease-progressing exRNAs in a bidirectional manner. As an example, subarachnoid hemorrhage (SAH), caused by the rupture of an intracranial aneurysm, not only contributes to hemorrhagic strokes to a large degree [248], to microglia accumulation and activation, but may also lead to a cascade of systemic pathologies, including cardiac damage after SAH (Xu et al., 2021, unpublished). In which way different types of disseminating exRNAs may causally contribute to the underlying pathology of the interplay between cerebral hemorrhage and cardiac dysfunction is poorly understood and requires intensive future studies.

Likewise, different types of exDNA such cfDNA or NETs have been identified under various pathophysiological conditions (including hyperinflammation, tumor progression or neurodegeneration) in the brain and can contribute to disease onset and progression in various ways [52, 210, 447]. A common denominator in the pathogenesis is the release of mtDNA and cfDNA, the latter being particularly available in NETs, whereby both parameters may serve as disease biomarkers in plasma and CSF as well. As to MS, oxidized exDNA together with oxidized lipids are highly enriched in lesions and plaques, to be associated with active demyelination and axonal or neuronal injury [141]. Despite the fact that the mechanisms leading to demyelination and neurodegeneration are poorly understood, mitochondrial damage together with the release of mtDNA as well as complex formation of extracellular nucleic acids with basic myelin sheet proteins may contribute to the initial patho-mechanisms of neurodegeneration and -regeneration in the cortex of patients with MS.

With regard to ischemic stroke, particularly cfDNAs do correlate with worse prognosis, whereas the depletion of platelets or platelet-specific DAMPs (as agonists for NETosis) greatly improved stroke outcomes. Here, the administration of a novel endogenous poly-peptide, designated neonatal NET-inhibitory factor (nNIF) [438], in experimental stroke models improved long-term neurological and motor function as well as enhanced survival after stroke [80]. nNIF specifically blocked NET formation without affecting neutrophil recruitment after stroke. Together with the combined application of DNase1/t-PA, as described in this article, nNIF and related peptides may serve as new therapeutic regimen towards NETs as targets in ischemic stroke. Interestingly enough, the generation of NETs in association with SAH could be prevented by administration of either RNase A or DNase1, an observation that indicates a close association between different SENAs in situations of brain tissue damage [119, 444]. Despite the fact that DNase-related targeting also of other NET-associated pathologies such as cystic fibrosis, sepsis or subcutaneous lupus erythematosus may provide some benefit, the patho-mechanistic role of extracellular cytotoxic histones (as a major component of NETs) in these diseases as well as in brain-associated pathologies requires further engaged investigation to weaken the dark side of NETs [328].

## Conclusions

In recent years, we have witnessed the appearance and molecular interplay of the class of diverse extracellular nucleic acid molecules, exRNAs and exDNAs, present in different body fluids and tissues, including their structural characterization, biological functions and possible medical applications. A plethora of basic research as well as clinical studies concerned with the profiling of these high and low molecular weight poly-anionic compounds, either in isolated form, as complexes with proteins or in association with EVs, has been undertaken. An emphasis was particularly laid on their definition as biomarkers such as for miRNAs, mtDNAs or NET fragments (summarized in Tables 3, 4, 5, 6) and their potential as causal disease factors, especially for neuroinflammatory and neurodegenerative disorders as discussed here.

Although we are far away from understanding all the mechanistic details in the biological systems we have touched upon in this review, we certainly appreciate the diversity of molecular interactions by which such nucleic acid DAMPs provide ways to provoke cellular signal transduction pathways, both under physiological as well as pathological conditions. Based on such data, new antagonistic approaches have been suggested (including the treatment with RNase or DNase, antagomirs and others) to combat certain neurological disorders (summarized in Table 2). Whether such regimen will find their way into the broad medical field remains to be seen, at least some miRNA-based biomarkers appear to be used in diagnostic analysis.

Nevertheless, the topic addressed in this review is often underappreciated and dismissed as a normal consequence of cell stress, tissue injury, infection, or the healing process in the brain, but has potent translational impact for medical applications in neuroinflammatory and neurodegenerative diseases. A discussion on these disorders, exacerbated by extracellular nucleic acids, is virtually non-existent in the literature, and the here presented approaches may find their way into possible new treatments. It is hoped that this review would stimulate basic researchers as well as clinical scientists in their research fields devoted to neurological pathologies.

## Data Availability

Not applicable.

## Abbreviations

Aβ: Amyloid-β
AD: Alzheimer's disease
AIM2: Absent in melanoma
ALS: Amyotrophic lateral sclerosis
AMPK: AMP-activated kinase
ANXA2: Annexin A2
AP2B1: Adaptor-related protein complex 2 subunit β1
APC: Antigen-presenting cell
APP: Amyloid precursor protein
AUF1: AU-rich element/poly(U)-binding/degradation factor 1
AXIN1: Axis inhibition protein 1
BACE1: β-Site APP cleaving enzyme-1
BBB: Blood–brain barrier
BMI1: B lymphoma Mo-MLV insertion region 1 homolog
CaMKKβ: Calcium/calmodulin-dependent protein kinase kinase beta
CARD: Caspase activation and recruitment domain
cfDNA: Cell-free DNA
cGAS: Cyclic GMP/AMP (cGAMP) synthase
circRNA: Circular RNA
CNS: Central nervous system
CSF: Cerebrospinal fluid
CREB: CAMP response element-binding protein
DAMPs: Danger-associated molecular patterns
dsDNA: Double-stranded DNA
dsRNA: Double-stranded RNA
EAE: Experimental autoimmune encephalomyelitis
ET: Extracellular trap
EV: Extracellular vesicle
FIRRE: Functional intergenic RNA repeat element
FUS: Fused in sarcoma
GAS5: Growth arrest-specific 5
GDF-15: Growth differentiation factor 15
HAGLROS: HAGLR opposite strand
HD: Huntington's disease
HDAC1: Histone deacetylase 1
HLA-DR: Human leukocyte antigen-DR isotype
HMGB1: High mobility group box protein 1
hnRNP K: Heterogeneous nuclear ribonucleoprotein K
HOTAIR: HOX transcript antisense RNA
IFN: Interferon
IKZF3: Ikaros family zinc finger protein 3
IL: Interleukin
iNOS: Inducible nitric oxide synthase
I/R: Ischemia/reperfusion
IRAK1: IL-1 receptor-associated kinase 1
LCP1: Lymphocyte cytosolic protein 1
LGP2: Laboratory of genetics and physiology 2
lincRNA-p21: Long intergenic noncoding RNA-p21
lncRNA: Long non-coding RNA
LPS: Lipopolysaccharide
LRRK2: Leucine rich repeat kinase 2
Maclpil: Macrophage contained LCP1 related pro-inflammatory lncRNA
MALAT1: Metastasis-associated lung adenocarcinoma transcript 1
MAF: Musculoaponeurotic fibrosarcoma
MAVS: Mitochondrial antiviral-signaling protein
MDA5: Melanoma differentiation-associated protein 5
mexDNA: Mitochondrial exDNA
MIB1: E3 Ubiquitin ligase Mindbomb1
miRNA: MicroRNA
MPP+: 1-Methyl-4-phenylpyridinium
MPTP: 1-Methyl-1-4-phenyl-1,2,3,6-tetrahydripyridine hydrochloride
mRNA: Messenger RNA
MS: Multiple sclerosis
mtDNA: Mitochondrial DNA
mTOR: Mechanistic target of rapamycin
MyD88: Myeloid differentiation factor 88
NBR1: Next to BRCA1 gene 1
ncRNA: Non-coding RNA
NEAT1: Nuclear paraspeckle assembly transcript 1
Nespas: Nesp-antisense
NETs: Neutrophil extracellular traps
NF-κB: Nuclear factor-kappa B
NFT: Intraneuronal neurofibrillary tangle
NLR: Nucleotide-binding oligomerization domain (NOD)-like receptor
NLRP3: NLR family pyrin domain containing protein 3
nucDNA: Nuclear DNA
OGD/R: Oxygen–glucose deprivation/reoxygenation
PAD4: Peptidyl-arginine-deiminase-4
PAMPs: Pathogen-associated molecular patterns
PBMC: Peripheral blood mononuclear cell
PD: Parkinson's disease
PINK1: PTEN-induced kinase 1
PPARγ: Peroxisome proliferator-activated receptor gamma
PPMS: Primary progressive MS
PRC2: Polycomb repressive complex 2
PRR: Pattern recognition receptor
RAGE: Receptor for advanced glycation end-products
rexRNA: Extracellular rRNA
RLR: Retinoic acid-inducible gene-1 (RIG-1)-like receptor
RMST: Rhabdomyosarcoma 2-associated transcript
RN7SK: RNA component of 7SK nuclear ribonucleoprotein
ROS: Reactive oxygen species
RRMS: Relapsing–remitting MS
rRNA: Ribosomal RNA
rt-PA: Recombinant tissue plasminogen activator
SAH: Subarachnoid hemorrhage
SFPQ: Splicing factor proline- and glutamine-rich
SHIP1: SH2 domain-containing inositol 5′-phosphatase 1
SIGMAR1: Sigma non-opioid intracellular receptor 1
SIRT1: Sirtuin 1
SLE: Systemic lupus erythematosus
SNCA: α-Synuclein
SNHG: Small nucleolar RNA host gene
SOCS: Suppressor of cytokine signaling
SPMS: Secondary progressive MS
ssRNA: Single-stranded RNA
STAT6: Signal transducer and activator of transcription 6
STING: Stimulator of interferon genes
TAK1: Transforming growth factor-β-activated kinase 1
tau: Tubulin associated unit
TBI: Traumatic brain injury
TBK1: TANK-binding kinase
TFAM: Mitochondrial transcription factor A
TIR: Toll/IL-1 receptor
TLR: Toll-like receptor
TNF-α: Tumor necrosis factor-α
TRAF6: TNF receptor-associated factor 6
TREM2: Triggering receptor expressed on myeloid cells 2
tRNA: Transfer RNA
TUG1: Taurine upregulated 1
UCHL1: Ubiquitin C-terminal hydrolase L1
ULK1: UNC51-like kinase-1
VEGF: Vascular endothelial growth factor
ZFAS1: ZNFX1 antisense RNA 1
